# Dynamical behavior of coal shearer under the influence of multiple factors in slant-cutting conditions

**DOI:** 10.1038/s41598-021-98049-x

**Published:** 2021-09-16

**Authors:** Xinwei Yang, Xianfeng Zou, Shuai Zhang, Hongyue Chen, Yajing Wei, Pengfei Li

**Affiliations:** 1 Faculty of Electrical and Control Engineering, Liaoning Technical University, Huludao, 125105 Liaoning China; 2 School of Mechanical Engineering, Liaoning Technical University, Fuxin, 123000 Liaoning China; 3 Research Institute of Technology and Equipment for the Exploitation and Utilization of Mineral Resources, Fuxin, 123000 Liaoning China; 4 Fuxin City Industrial Technology Research Institute, Fuxin, 123000 Liaoning China

**Keywords:** Mechanical engineering, Statistical physics, thermodynamics and nonlinear dynamics

## Abstract

Aiming at the problem of severe vibration and abnormal wear and tear of various components in coal shearer under slant-cutting conditions, a non-linear dynamics model with 13 degrees of freedom for a coal shearer under slant-cutting conditions is developed using vibration mechanics and multi-body dynamics theory, and the characteristics of the slide shoes-middle groove contact, the ranging arm-haulage unit connection with gaps and the guidance sliding boots-pin rail multi-surface contact with gaps are described based on three-dimensional fractal theory and Hertz contact theory. Based on Huco's law, the ranging arm and the hydraulic rod are assumed to be flexible beams, the rigidity characteristics of the ranging arm itself, the connection characteristics of the haulage unit and the fuselage are described, a drum correction load with a traction speed correction factor is proposed as the external excitation of the system, and the model is solved and analyzed. The research results show that the change of traction speed has a greater influence on the vibration swing angle and displacement of the front drum, front ranging arm and front walking unit, and the vibration swing angle and displacement of the three increase with the increase of traction speed, while the change of coalface hardness coefficient has less influence on the vibration displacement of the key components of the coal shearer. Under the working parameters of *v* = 3 m/min and *f* = 3, the swing angle and displacement of the front ranging arm and front drum fluctuate in the ranges of − 0.4–0.1 rad and – 15–15 mm respectively; the vibration acceleration is – 300–300 rad/s^2^ and – 200–200 mm/s^2^ respectively, the main vibration frequencies are 16.63 Hz and 12.14 Hz respectively, and finally the results are verified by experimental methods.

## Introduction

As a major source of energy, coal, with the increasing overall mining depth of coal mines, has put forward new and higher requirements for coal mining equipment and technology levels, as well as new challenges for production safety and production efficiency^1,2^. As an important piece of comprehensive mining equipment, the dynamics of the coal shearer affects the stability of the machine and, in turn, the overall safety and economic efficiency of the comprehensive mining operation. Especially under the influence of the impact of the big load of the drum under the slant-cutting condition, the coal shearer generates strong vibration, which aggravates the loss of key components, such as abnormal wear of the teeth of the transmission system, broken teeth of the drive wheel, uncoordinated matching between the walking units and the scraper conveyor, etc., reducing the service life of each component and increasing the maintenance time. The study of the dynamics of coal shearer under slant-cutting conditions is therefore of great importance in guiding the optimization of the operating parameters of coal shearer, fault diagnosis and improving the stability of coal shearer. At present, scholars have done some research work on the whole system of coal shearer and its key components. In the study of the transmission system of the cutting section, the dynamic characteristics of the ranging arm transmission system under the influence of factors such as ranging arm shell deformation and gear meshing temperature have been analyzed mainly by analytical method and analytical-finite element method^3–6^. By means of the obtained dynamics of the conventional system of the ranging arm in particular, Jiang et al.^7^ further designed a control method for suppressing large amplitude and unstable vibrations of the drive train based on the Routh-Hurwitz stability criterion; Chen et al.^8^ for the chaotic motion generated by the system and proposed to excite the unsteady motion of the control system by applying periodic resonance; Qin et al.^9^ optimized the topology of the ranging arm housing and analyzed the effect of the optimized deformed housing on the dynamic characteristics of the drive train; Zeng et al.^10^ analyzed and studied the dynamics of the coupling between the cutting force of the drum and the height-adjusting hydraulic system using a joint simulation technique; In the study of the haulage unit drive system, Zhang et al.^11^ proposed a drum load model for slant-cutting conditions, and the dynamics model of the haulage unit drive system is solved and analyzed; Zhou et al.^12^ used the saddle point approximation method to assess the maximum contact stress of the drive train and analyzed the reliability of the planetary gear system of coal shearer; Dejian et al.^13^ analyzed the drive characteristics and the dynamics of the haulage unit of a coal shearer with different operating parameters under the influence of longitudinal sway; Zhou et al.^14^ proposed a method for assessing the reliability of haulage unit drive systems and reliability-based sensitivity analysis and analyzed the planetary gear mechanism in the drive train; for the dynamics analysis of the whole system, Hongyue et al.^15^ used virtual technology to simulate and analyze the support characteristics of the four slide shoes of the coal shearer under different working conditions; Zhang et al.^16^ used Workbench finite element simulation software to analyze the modal and pre-stress constrained model of a three-drum coal shearer; Xinwei et al.^17^ used a combination of numerical solution and experimental verification to analyze the dynamics of the coupled traction-swing of a coal shearer under multiple factors and operating conditions. Boloz et al.^18–20^ used an experimental method to obtain the working parameters of various equipment at the comprehensive mining operation and used a numerical solution to obtain the effect of different structures for coal shearer drum and different arrangements of picks on the cutting efficiency.

In the above studies, only the dynamic characteristics of a single system or a single direction of the coal shearer have been analyzed, while the vibration characteristics of the components of the coal shearer, as a large and complex mechanical equipment, are affected by each other, and there is less research on the dynamic characteristics of the coal shearer under slant-cutting working conditions. To address the above problems, this article adopts the multi-body dynamics theory to establish the dynamics model of the coal shearer with 13 degrees of freedom under the slant-cutting condition, describes the connection rigidity of each key component and the contact rigidity with the scraper conveyor, proposes a modified drum load as the external excitation of the system, solves for each key component of the coal shearer under different working parameters, and finally uses the experimental method to verify the accuracy of the solution results. The results of the study provide a theoretical basis for the reliability of the complete coal shearer and the prediction of the fatigue life of key components.

## Modeling the dynamics of coal shearer under slant-cutting conditions

**Figure 1:**
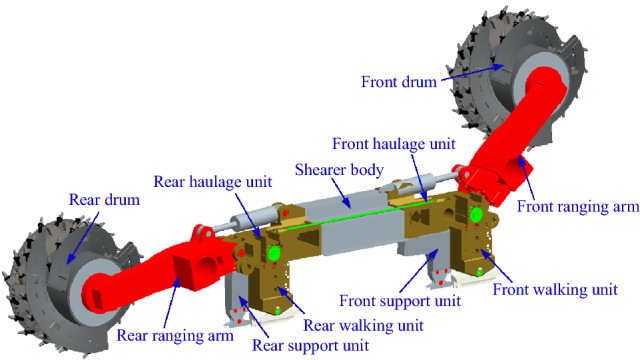
Three-dimensional schematic of the coal shearer.

**Figure 2:**
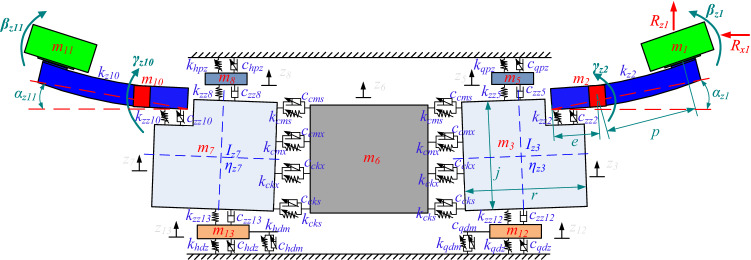
Non-linear dynamics model of a coal shearer under slant-cutting conditions.

Figure 1 shows a three-dimensional schematic of the coal shearer. Due to the complex structure of the whole coal shearer, the model is suitably simplified considering the feasibility of the solution and the accuracy of the results. As shown in Fig. 2, the concentrated mass method is used to divide the coal shearer into front and rear drums, front and rear ranging arms, front and rear haulage units, front and rear walking units, front and rear support units and the shearer body, with a total of 11 sections, and assuming that: (1) the mass of each part is concentrated at the center of gravity of each part; (2) the influence of the hydraulic system, the electrical system and the transmission system on the dynamic characteristics of the whole machine is ignored; (3) the front and rear ranging arms are massless beams at the front section of the center of gravity and the rear section is rigid; (4) the contact and connection between the parts are described using rigidity and resistances elements; (5) the gap between the two sides of the connection position of each part in the initial position is equal. Based on the above assumptions, a model of coal shearer dynamics under slant-cutting conditions is established and its variable annotated table is shown in Table 1.

1. Kinetic energy of the system:
  1 $$ \begin{aligned} T & = T_{1} + T_{2} + T_{3} + T_{5} + T_{12} + T_{6} + T_{7} + T_{13} + T_{8} + T_{10} + T_{11} \hfill \\ & = \frac{1}{2}m_{1} \left( {v_{x1}^{2} + v_{z1}^{2} } \right) + \frac{1}{2}m_{2} \left( {v_{x2}^{2} + v_{z2}^{2} } \right) + \frac{1}{2}m_{3} \dot{z}_{3}^{2} + \frac{1}{2}I_{z3} \cdot \dot{\eta }_{z3}^{2} + \frac{1}{2}m_{12} \dot{z}_{12}^{2} + \frac{1}{2}m_{5} \dot{z}_{5}^{2} + \frac{1}{2}m_{6} \dot{z}_{6}^{2} \hfill \\ & \quad + \frac{1}{2}m_{7} \dot{z}_{7}^{2} + \frac{1}{2}I_{z7} \cdot \dot{\eta }_{z7}^{2} + \frac{1}{2}m_{13} \dot{z}_{13}^{2} + \frac{1}{2}m_{8} \dot{z}_{8}^{2} + \frac{1}{2}m_{10} \left( {v_{x10}^{2} + v_{z10}^{2} } \right) + \frac{1}{2}m_{11} \left( {v_{x11}^{2} + v_{z11}^{2} } \right) \hfill \\ \end{aligned} $$
  Where *v*_x1_、*v*_z1_ are the front drum traction direction and axial vibration speed respectively, *v*_x2_、*v*_z2_ are the front ranging arm traction direction and longitudinal vibration speed respectively.

**Table 1:** Description of symbols in Fig. 1.

Symbol	Explanation
*m*_1_, *m*_11_	The masses of the front and rear drums
*m*_2_, *m*_10_	The masses of the front and rear ranging arms
*m*_3_, *m*_7_	The masses of the front and rear haulage units
*m*_12_, *m*_13_	The masses of the front and rear walking units
*m*_5_, *m*_8_	The masses of the front and rear supporting units
*m*_6_	The mass of the shearer body
*z*_3_, *z*_7_	The vibration displacements of the front and rear haulage units
*z*_5_, *z*_8_	The vibration displacements of the front and rear supporting units
*z*_12_, *z*_13_	The vibration displacements of the front and rear walking units
*z*_6_	The vibration displacement of the shearer body
*I*_z3_, *I*_z7_	The rotational inertia of the front and rear haulage unit
*η*_z3_, *η*_z7_	The vibration swings of the front and rear haulage unit
*α*_z1_, *α*_z11_	Initial angle of the front and rear ranging arms to the haulage unit
*β*_z1_, *β*_z11_	The vibration swings of the front and rear drums
*γ*_z2_, *γ*_z10_	The vibration swings of the front and rear ranging arms
*R*_x1_, *R*_z1_	Cutting load on the front drum in the traction direction and axial direction
*k*_z2_, *k*_z10_	Equivalent rigidities of the front and rear ranging arms
*k*_zz2_, *c*_zz2_, *k*_zz10_, *c*_zz10_	Front and rear ranging arms- haulage unit joint rigidities and resistances
*k*_zz5_, *c*_zz5_, *k*_zz8_, *c*_zz8_	Front and rear supporting units—haulage unit joint rigidities and resistances
*k*_zz12_、*c*_zz12_、*k*_zz13_, *c*_zz13_	Front and rear walking units—haulage unit joint rigidities and resistances
*k*_qpz_, *c*_qpz_, *k*_hpz_, *c*_hpz_	Front and rear supporting units—middle groove joint rigidities and resistances
*k*_qdz_, *c*_qdz_, *k*_hdz_, *c*_hdz_	Front and rear guidance sliding boots inner face-pin rail contact rigidities and resistances
*k*_qdm_, *c*_qdm_, *k*_hdm_, *c*_hdm_	Front and rear guidance sliding boots inner top face-pin rail contact rigidities and resistances
*k*_cms_, *c*_cms_, *k*_cmx_, *c*_cmx_	The equivalent rigidities and resistances of the upper and lower hydraulic rods on the coalface side
*k*_cks_, *c*_cks_, *k*_ckx_, *c*_ckx_	The equivalent rigidities and resistances of the upper and lower hydraulic rods on the goaf side
*e*	The radius of gyration of the gravity center of the ranging arm
*p*	The distance between the gravity centers of the drum and ranging arm
*j*	THE width of the haulage unit
*r*	the length of the haulage unit
  During normal operation of the coal shearer, the vibration swings of the front and rear ranging arms is small, then there is:
  $$ \left\{ \begin{aligned} v_{x2} & = e \cdot \dot{\gamma }_{z2} \cdot \cos \alpha_{z1} \hfill \\ v_{z2} & = \dot{z}_{3} + e \cdot \dot{\gamma }_{z2} \cdot \sin \alpha_{z1} \hfill \\ v_{x10} & = e \cdot \dot{\gamma }_{z10} \cdot \cos \alpha_{z11} \hfill \\ v_{z10} & = \dot{z}_{7} + e \cdot \dot{\gamma }_{z10} \cdot \sin \alpha_{z11} \hfill \\ \end{aligned} \right. \Rightarrow \left\{ \begin{aligned} v_{x1} & = e \cdot \dot{\gamma }_{z2} \cdot \cos \alpha_{z1} + p \cdot \dot{\beta }_{z1} \cdot \cos \alpha_{z1} \hfill \\ v_{z1} & = \dot{z}_{3} + e \cdot \dot{\gamma }_{z2} \cdot \sin \alpha_{z1} + p \cdot \dot{\beta }_{z1} \cdot \sin \alpha_{z1} \hfill \\ v_{x11} & = e \cdot \dot{\gamma }_{z10} \cdot \cos \alpha_{z11} + p \cdot \dot{\beta }_{z11} \cdot \cos \alpha_{z11} \hfill \\ v_{z11} & = \dot{z}_{7} + e \cdot \dot{\gamma }_{z10} \cdot \sin \alpha_{z11} + p \cdot \dot{\beta }_{z11} \cdot \sin \alpha_{z11} \hfill \\ \end{aligned} \right. $$
2. Potential energy of the system:
  2 $$ \begin{aligned} U & = \frac{1}{2}k_{z2} \left( {p \cdot \beta_{z1} } \right)^{2} + \frac{1}{2}k_{zz2} \left[ {e \cdot \gamma_{z2} - \left( {z_{3} + \frac{1}{2}r \cdot \eta_{z3} } \right) - \frac{{d_{xy} }}{2}} \right]^{2} + \frac{1}{2}k_{zz5} \left( {z_{5} - z_{3} - \frac{{d_{xp} }}{2}} \right)^{2} \hfill \\ & \quad + \frac{1}{2}k_{qpz} z_{5}^{2} + \frac{1}{2}k_{zz12} \left( {z_{3} - z_{12} - \frac{{d_{xd} }}{2}} \right)^{2} + \frac{1}{2}k_{qdz} \left( {z_{12} - \frac{{w_{xd} }}{2}} \right)^{2} + \frac{1}{2}k_{qdm} z_{12}^{2} \hfill \\ & \quad + \frac{1}{2}k_{cms} \left( {z_{3} + \frac{1}{2}l_{cms} \cdot \eta_{z3} + z_{7} + \frac{1}{2}l_{cms} \cdot \eta_{z7} - z_{6} } \right)^{2} + \frac{1}{2}k_{cmx} \left( {z_{3} + \frac{1}{2}l_{cmx} \cdot \eta_{z3} + z_{7} + \frac{1}{2}l_{cmx} \cdot \eta_{z7} - z_{6} } \right)^{2} \hfill \\ & \quad + \frac{1}{2}k_{cks} \left( {z_{3} + \frac{1}{2}l_{cks} \cdot \eta_{z3} + z_{7} + \frac{1}{2}l_{cks} \cdot \eta_{z7} - z_{6} } \right)^{2} + \frac{1}{2}k_{ckx} \left( {z_{3} + \frac{1}{2}l_{ckx} \cdot \eta_{z3} + z_{7} + \frac{1}{2}l_{ckx} \cdot \eta_{z7} - z_{6} } \right)^{2} \hfill \\ & \quad + \frac{1}{2}k_{hdm} z_{12}^{2} + \frac{1}{2}k_{hdz} \left( {z_{13} - \frac{{w_{xd} }}{2}} \right)^{2} + \frac{1}{2}k_{zz13} \left( {z_{7} - z_{13} - \frac{{d_{xd} }}{2}} \right)^{2} + \frac{1}{2}k_{hpz} z_{8}^{2} + \frac{1}{2}k_{zz8} \left( {z_{8} - z_{7} - \frac{{d_{xp} }}{2}} \right)^{2} \hfill \\ & \quad + \frac{1}{2}k_{zz10} \left[ {e \cdot \gamma_{z10} - \left( {z_{7} + \frac{1}{2}r \cdot \eta_{z7} } \right) - \frac{{d_{xy} }}{2}} \right]^{2} + \frac{1}{2}k_{z10} \left( {p \cdot \beta_{z11} } \right)^{2} \hfill \\ \end{aligned} $$
3. Dissipated energy of the system:
  3 $$ \begin{aligned} D & = \frac{1}{2}c_{zz2} \left[ {e \cdot \dot{\gamma }_{z2} - \left( {\dot{z}_{3} + \frac{1}{2}r \cdot \dot{\eta }_{z3} } \right)} \right]^{2} + \frac{1}{2}c_{zz5} \left( {\dot{z}_{5} - \dot{z}_{3} } \right)^{2} + \frac{1}{2}c_{qpz} \dot{z}_{5}^{2} + \frac{1}{2}c_{zz12} \left( {\dot{z}_{3} - \dot{z}_{12} } \right)^{2} \hfill \\ & \quad + \frac{1}{2}c_{qdz} \dot{z}_{12}^{2} + \frac{1}{2}c_{qdm} \dot{z}_{12}^{2} + \frac{1}{2}c_{cms} \left( {\dot{z}_{3} + \frac{1}{2}l_{cms} \cdot \dot{\eta }_{z3} + \dot{z}_{7} + \frac{1}{2}l_{cms} \cdot \dot{\eta }_{z7} - \dot{z}_{6} } \right)^{2} \hfill \\ & \quad + \frac{1}{2}c_{cmx} \left( {\dot{z}_{3} + \frac{1}{2}l_{cmx} \cdot \dot{\eta }_{z3} + \dot{z}_{7} + \frac{1}{2}l_{cmx} \cdot \dot{\eta }_{z7} - \dot{z}_{6} } \right)^{2} + \frac{1}{2}c_{cks} \left( {\dot{z}_{3} + \frac{1}{2}l_{cks} \cdot \dot{\eta }_{z3} + \dot{z}_{7} + \frac{1}{2}l_{cks} \cdot \dot{\eta }_{z7} - \dot{z}_{6} } \right)^{2} \hfill \\ & \quad + \frac{1}{2}c_{ckx} \left( {\dot{z}_{3} + \frac{1}{2}l_{ckx} \cdot \dot{\eta }_{z3} + \dot{z}_{7} + \frac{1}{2}l_{ckx} \cdot \dot{\eta }_{z7} - \dot{z}_{6} } \right)^{2} + \frac{1}{2}c_{hdm} \dot{z}_{12}^{2} + \frac{1}{2}c_{hdz} \dot{z}_{13}^{2} \hfill \\ & \quad + \frac{1}{2}c_{zz13} \left( {\dot{z}_{7} - \dot{z}_{13} } \right)^{2} + \frac{1}{2}c_{hpz} \dot{z}_{8}^{2} + \frac{1}{2}c_{zz8} \left( {\dot{z}_{8} - \dot{z}_{7} } \right)^{2} + \frac{1}{2}c_{zz10} \left[ {e \cdot \dot{\gamma }_{z10} - \left( {\dot{z}_{7} + \frac{1}{2}r \cdot \dot{\eta }_{z7} } \right)} \right]^{2} \hfill \\ \end{aligned} $$
  Substitute Eqs. (1), (2) and (3) into the Lagrnge kinetic equation in equation
  4 $$ \frac{d}{dt}\left( {\frac{\partial T}{{\partial \dot{q}_{i} }}} \right) - \frac{\partial T}{{\partial q_{i} }} + \frac{\partial U}{{\partial q_{i} }} + \frac{\partial D}{{\partial \dot{q}_{i} }} = F_{i} \begin{array}{*{20}c} {} & {} \\ \end{array} \left( {i = 1,2, \ldots ,n} \right) $$
  where *F*_*i*_ is the generalized force acting along the direction of the generalized coordinate *qi*.
  Collation gives:
  5 $$ {\varvec{M\ddot{Z}}} + {\varvec{C\dot{Z}}} + {\varvec{KZ}} = {\varvec{F}} $$
  See Appendix for matrices ***M***, ***C***, ***K***, ***Z***, ***F***.

## Modeling the rigidity between key components

### Model for the tangential rigidity of the joint between the slide shoes and the middle groove

There is no ideal smooth and rigid surface on the microscopic level; there are countless micro-convex bodies of varying sizes and shapes on the surface. During contact and collision, the micro-convex body is deformed, which affects the contact characteristics of the bonding surface and the dynamics of the mechanical system. In the following study, the effect of the elastic deformation of the micro-convex body on the contact properties of the bonding surface is considered.

**Figure 3:**
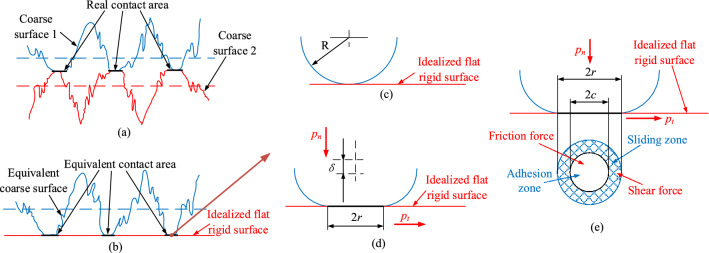
Schematic diagram of the contact between the slide shoes and the middle groove: (**a**) actual contact interface, (**b**) equivalent contact interface, (**c**) contact before a load is applied, (**d**) contact when a load is applied, (**e**) contact between an asperity and the idealized surface with friction considered.

Figure 3 shows a schematic diagram of the contact between the slide shoes and the middle groove, where Fig. 3a shows the actual contact state between the slide shoes and the middle groove. During the contact between the slide shoes and the middle groove, the actual contact and action of the micro-convex bodies on the surface of the two parts is made. Based on GW and CEB contact theory^21,22^, the problem of contact between a slide shoes and a middle groove is assumed to be a problem of contact between a rough surface and an ideally smooth surface, whose microscopic contact state is shown in Fig. 3b. For a single micro-convex body on the equivalent contact region of Fig. 3b, it can be approximated as a sphere. The contact state when not subjected to a load is shown in Fig. 3c and the contact state when subjected to a normal load *p*_*n*_ is shown in Fig. 3d. When the tangential load *p*_*t*_ between the slide shoes and the middle groove is greater than the maximum static friction, the bonding surface only appears to be in full slip, as shown in Fig. 3e. The individual micro-convex contact areas in the slide shoes and middle groove bonding surface can be divided into an adhesion area and a sliding area.

Based on the GW and CEB contact models^18,19^, the tangential contact rigidity between the front and rear slide shoes and the middle groove is:

6 $$ k_{qpz} = \left( {\frac{3}{4\pi }} \right)^{\frac{1}{3}} \cdot \frac{4D}{{1 - D}} \cdot \frac{{\frac{{G_{s} \cdot P_{tqp} }}{{\mu P_{nqp} }}}}{{1 - \left( {1 - \frac{{P_{tqp} }}{{\mu P_{nqp} }}} \right)^{\frac{2}{3}} }} \cdot \left( {\frac{{P_{nqp} }}{{E_{pz} \cdot A_{pz} }}} \right) \cdot \psi^{1 - 0.5D} G^{{\frac{1 - D}{3}}} a_{\max }^{0.5D} \left( {a_{pz}^{{\frac{1 - D}{3}}} - a_{\mu c}^{{\frac{1 - D}{3}}} } \right) $$

7 $$ k_{hpz} = \left( {\frac{3}{4\pi }} \right)^{\frac{1}{3}} \cdot \frac{4D}{{1 - D}} \cdot \frac{{\frac{{G_{s} \cdot P_{thp} }}{{\mu P_{nqp} }}}}{{1 - \left( {1 - \frac{{P_{thp} }}{{\mu P_{nhp} }}} \right)^{\frac{2}{3}} }} \cdot \left( {\frac{{P_{nhp} }}{{E_{pz} \cdot A_{pz} }}} \right) \cdot \psi^{1 - 0.5D} G^{{\frac{1 - D}{3}}} a_{\max }^{0.5D} \left( {a_{pz}^{{\frac{1 - D}{3}}} - a_{\mu c}^{{\frac{1 - D}{3}}} } \right) $$

8 $$ E_{pz} = \left( {\frac{{1 - \upsilon_{p}^{2} }}{{E_{p} }} + \frac{{1 - \upsilon_{z}^{2} }}{{E_{z} }}} \right)^{ - 1} $$

where *D* is the fractal dimension; *G*_*s*_ is the equivalent shear modulus of a single micro-convex body; *μ* is the friction coefficient; and *G* is the fractal roughness parameter^23^; *ψ* is the expansion factor of the contact size distribution of the micro-convex body (*ψ* > 1) and its value is related to the fractal dimension *D*^24^; *E*_*pz*_ is the equivalent elastic modulus of the slide shoes-middle groove; *E*_*p*_ and *E*_*z*_ are the elastic modulus of the slide shoes and the middle groove respectively; *υ*_*p*_ and *υ*_*z*_ are the Poisson's ratios of the slide shoes and the middle groove respectively; *P*_*tqp*_ and *P*_*thp*_ are the tangential loads on the front and rear slide shoes and the middle groove union surfaces respectively; *P*_*nqp*_ and *P*_*nhp*_ are the normal loads on the front and rear slide shoes and middle groove union surfaces respectively; *a*_*pz*_ is the actual contact area between the slide shoes and the middle groove; *a*_*pz*_ is the actual contact area between the slide shoes and the micro-convex body of the middle groove; *a*_max_ is the maximum area of contact of a single micro-convex body on the joint surface of the slide shoes and the middle groove; *a*_*μc*_ is the elastic–plastic critical contact area of the micro-convex body at the bonding surface of the slide shoes and the middle groove.

### Normal rigidity model for the ranging arm and haulage unit joint with gap

**Figure 4:**
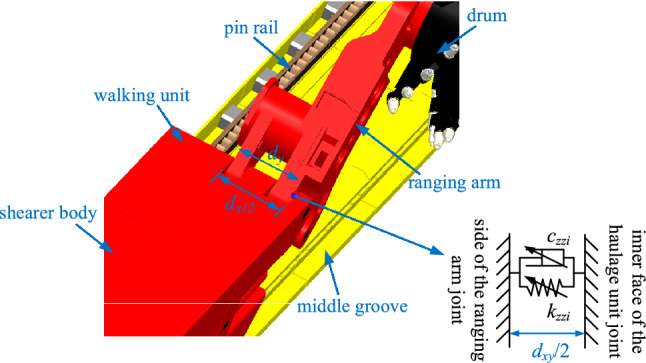
Schematic diagram of the connection between the ranging arm and the haulage unit.

As the contact characteristics between the ranging arm and the haulage unit are mainly influenced by the gap between the ranging arm and the haulage unit under slant-cutting conditions, the rigidity model of the ranging arm-haulage unit connection under the influence of the gap is described below. Figure 4 shows a schematic diagram of the connection between the ranging arm and the haulage unit, where *d*_*x*12_ is the total width at the connection of the haulage unit to the ranging arm, *d*_*y*_ is the total width of the hinge lug connecting the ranging arm to the haulage unit, *d*_*xy*_ = *d*_*x*12_-*d*_*y*_ is the gap between the ranging arm and the haulage unit connection.

The front and rear ranging arms are in contact with the haulage unit under gap conditions for the following four conditions:

1. When no contact collision has occurred between either the front or rear ranging arm and the haulage unit connection:
  $$ \left\{ \begin{gathered} \left| { - \left[ {e \cdot \sin \left( {\gamma_{z2} + \alpha_{z1} } \right)} \right] + z_{3} + \sin \eta_{z3} \cdot r/2} \right| \le d_{xy} /2 \hfill \\ \left| { - \left[ {e \cdot \sin \left( {\gamma_{z10} + \alpha_{z11} } \right)} \right] + z_{7} + \sin \eta_{z7} \cdot r/2} \right| \le d_{xy} /2 \hfill \\ \end{gathered} \right. \Rightarrow \left\{ \begin{gathered} k_{zz2} = 0 \hfill \\ k_{zz10} = 0 \hfill \\ \end{gathered} \right. $$
2. When a contact collision has occurred between the front ranging arm and the haulage unit connection, and when no contact collision has occurred between the rear ranging arm and the haulage unit connection:
  $$ \left\{ \begin{gathered} \left| { - \left[ {e \cdot \sin \left( {\gamma_{z2} + \alpha_{z1} } \right)} \right] + z_{3} + \sin \eta_{z3} \cdot r/2} \right| > d_{xy} /2 \hfill \\ \left| { - \left[ {e \cdot \sin \left( {\gamma_{z10} + \alpha_{z11} } \right)} \right] + z_{7} + \sin \eta_{z7} \cdot r/2} \right| \le d_{xy} /2 \hfill \\ \end{gathered} \right. \Rightarrow \left\{ \begin{gathered} k_{zz2} \ne 0 \hfill \\ k_{zz10} = 0 \hfill \\ \end{gathered} \right. $$
3. When no contact collision has occurred between the front ranging arm and the haulage unit connection, and when a contact collision has occurred between the rear ranging arm and the haulage unit connection:
  $$ \left\{ \begin{gathered} \left| { - \left[ {e \cdot \sin \left( {\gamma_{z2} + \alpha_{z1} } \right)} \right] + z_{3} + \sin \eta_{z3} \cdot r/2} \right| \le d_{xy} /2 \hfill \\ \left| { - \left[ {e \cdot \sin \left( {\gamma_{z10} + \alpha_{z11} } \right)} \right] + z_{7} + \sin \eta_{z7} \cdot r/2} \right| > d_{xy} /2 \hfill \\ \end{gathered} \right. \Rightarrow \left\{ \begin{gathered} k_{zz2} = 0 \hfill \\ k_{zz10} \ne 0 \hfill \\ \end{gathered} \right. $$
4. When a contact collision has occurred between both the front and rear ranging arm and the haulage unit connection:
  $$ \left\{ \begin{gathered} \left| { - \left[ {e \cdot \sin \left( {\gamma_{z2} + \alpha_{z1} } \right)} \right] + z_{3} + \sin \eta_{z3} \cdot r/2} \right| > d_{xy} /2 \hfill \\ \left| { - \left[ {e \cdot \sin \left( {\gamma_{z10} + \alpha_{z11} } \right)} \right] + z_{7} + \sin \eta_{z7} \cdot r/2} \right| > d_{xy} /2 \hfill \\ \end{gathered} \right. \Rightarrow \left\{ \begin{gathered} k_{zz2} \ne 0 \hfill \\ k_{zz10} \ne 0 \hfill \\ \end{gathered} \right. $$
  Combining the four contact cases above, the front and rear ranging arm contact rigidity with the haulage unit is:
  9 $$ \left\{ {\begin{array}{*{20}c} \begin{gathered} k_{zz2} = \frac{{2E_{xy} D}}{{\sqrt \pi \left( {1 - D} \right)}}\psi^{{\left( {2 - D} \right)/2}} \left( {z_{3} + \frac{r}{2} \cdot \sin \eta_{3} - \left[ {e \cdot \sin \left( {\gamma_{z2} + \alpha_{z1} } \right)} \right] - \frac{{d_{xy} }}{2}} \right)^{D/2} \hfill \\ \cdot \left[ {\left( {z_{3} + \frac{r}{2} \cdot \sin \eta_{3} - \left[ {e \cdot \sin \left( {\gamma_{z2} + \alpha_{z1} } \right)} \right] - \frac{{d_{xy} }}{2}} \right)^{{\left( {1 - D} \right)/2}} - \left( {\frac{{a_{\mu xy} }}{2}} \right)^{{\left( {1 - D} \right)/2}} } \right] \hfill \\ \end{gathered} & {} & {\left| {z_{3} + \frac{r}{2} \cdot \sin \eta_{3} - \left[ {e \cdot \sin \left( {\gamma_{z2} + \alpha_{z1} } \right)} \right]} \right| > \frac{{d_{xy} }}{2}} \\ {k_{zz2} = 0} & {} & {\left| {z_{3} + \frac{r}{2} \cdot \sin \eta_{3} - \left[ {e \cdot \sin \left( {\gamma_{z2} + \alpha_{z1} } \right)} \right]} \right| \le \frac{{d_{xy} }}{2}} \\ \end{array} } \right. $$
  10 $$ \left\{ {\begin{array}{*{20}c} \begin{gathered} k_{zz10} = \frac{{2E_{xy} D}}{{\sqrt \pi \left( {1 - D} \right)}}\psi^{{\left( {2 - D} \right)/2}} \left( {z_{7} + \frac{r}{2} \cdot \sin \eta_{7} - \left[ {e \cdot \sin \left( {\gamma_{z10} + \alpha_{z11} } \right)} \right] - \frac{{d_{xy} }}{2}} \right)^{D/2} \hfill \\ \cdot \left[ {\left( {z_{7} + \frac{r}{2} \cdot \sin \eta_{7} - \left[ {e \cdot \sin \left( {\gamma_{z10} + \alpha_{z11} } \right)} \right] - \frac{{d_{xy} }}{2}} \right)^{{\left( {1 - D} \right)/2}} - \left( {\frac{{a_{\mu xy} }}{2}} \right)^{{\left( {1 - D} \right)/2}} } \right] \hfill \\ \end{gathered} & {} & {\left| {z_{7} + \frac{r}{2} \cdot \sin \eta_{7} - \left[ {e \cdot \sin \left( {\gamma_{z10} + \alpha_{z11} } \right)} \right]} \right| > \frac{{d_{xy} }}{2}} \\ {k_{zz10} = 0} & {} & {\left| {z_{7} + \frac{r}{2} \cdot \sin \eta_{7} - \left[ {e \cdot \sin \left( {\gamma_{z10} + \alpha_{z11} } \right)} \right]} \right| \le \frac{{d_{xy} }}{2}} \\ \end{array} } \right. $$
  where *E*_*xy*_ is the equivalent modulus of elasticity of the ranging arm-haulage unit; *a*_*pz*_ is the actual contact area between the slide shoes and the micro-convex body of the middle groove; *a*_*μxy*_ is the elastic–plastic critical contact area of the micro-convex body at the joint surface of the ranging arm and the haulage unit.

### Multi-surface contact rigidity model for guidance sliding boots and pin rail with a gap

**Figure 5:**
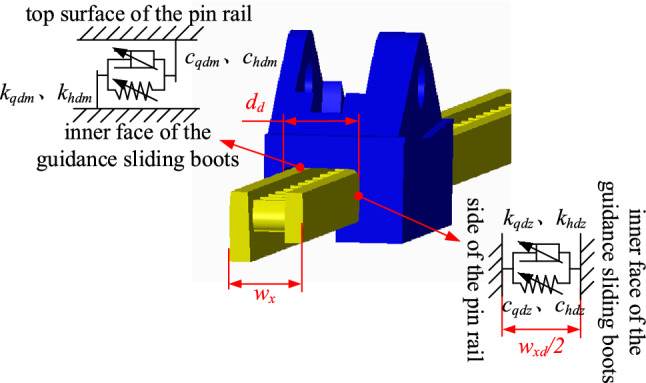
Schematic diagram of the guidance sliding boots in contact with the pin rail.

Based on the structure of the coal shearer and scraper conveyor, as shown in Fig. 5, there are both tangential and normal rigidity in the combination of the guidance sliding boots and the pin rail, which need to be described separately. Where *d*_*d*_ is the width of the inside of the guiding slide shoe of the coal shearer; *w*_*x*_ is the width of the pin rail; The gap between the guidance sliding boots and the side of the pin rail is *w*_*xd*_ = *d*_*d*_-*w*_*x*_*.*

Based on the method of describing the tangential rigidity of the slide shoes and the middle groove, the tangential rigidity of the combination of the top surface of the pin rail and the guidance sliding boots can be obtained as:

11 $$ k_{qdm} = \left( {\frac{3}{4\pi }} \right)^{\frac{1}{3}} \cdot \frac{4D}{{1 - D}} \cdot \frac{{\frac{{G_{dxs} \cdot P_{tqd} }}{{\mu P_{nqd} }}}}{{1 - \left( {1 - \frac{{P_{tqd} }}{{\mu P_{nqd} }}} \right)^{\frac{2}{3}} }} \cdot \left( {\frac{{P_{nqd} }}{{E_{dx} \cdot A_{dx} }}} \right) \cdot \psi^{1 - 0.5D} G^{{\frac{1 - D}{3}}} a_{dx\max }^{0.5D} \left( {a_{dx}^{{\frac{1 - D}{3}}} - a_{dx\mu c}^{{\frac{1 - D}{3}}} } \right) $$

12 $$ k_{hdm} = \left( {\frac{3}{4\pi }} \right)^{\frac{1}{3}} \cdot \frac{4D}{{1 - D}} \cdot \frac{{\frac{{G_{dxs} \cdot P_{thd} }}{{\mu P_{nhd} }}}}{{1 - \left( {1 - \frac{{P_{thd} }}{{\mu P_{nhd} }}} \right)^{\frac{2}{3}} }} \cdot \left( {\frac{{P_{nhd} }}{{E_{dx} \cdot A_{dx} }}} \right) \cdot \psi^{1 - 0.5D} G^{{\frac{1 - D}{3}}} a_{dx\max }^{0.5D} \left( {a_{dx}^{{\frac{1 - D}{3}}} - a_{dx\mu c}^{{\frac{1 - D}{3}}} } \right) $$

where *P*_*tqd*_ and *P*_*thd*_ are the tangential loads between the front and rear guidance sliding boots and the top surface of the pin rail respectively; *P*_*nqd*_ and *P*_*nhd*_ are the normal loads on the sides of the front and rear guidance sliding boots and pin rails respectively; *G*_*dxs*_ is the equivalent shear modulus of the micro-convex body at the joint of the guidance sliding boots and the pin rail; *E*_*dx*_ is the equivalent modulus of elasticity of the micro-convex body at the joint of the guidance sliding boots and the pin rail; *A*_*dx*_ is the actual contact area between the guidance sliding boots and the pin rail; *a*_*dx*max_ is the maximum contact area between the guidance sliding boots and the micro-convex body of the pin rail; *a*_*dx*_ is the actual contact area between the guidance sliding boots and the micro-convex body of the pin rail; *a*_*dxμc*_ is the elastic–plastic critical contact area of the micro-convex body of the guidance sliding boots to the pin rail.

There are four types of contact between the front and rear guidance sliding boots and the side of the pin rail under gap conditions as follows:

1. When no contact collision has occurred between either the front or rear guidance sliding boots and the side of the pin rail:
  $$ \left\{ \begin{gathered} \left| {z_{12} } \right| \le w_{xd} /2 \hfill \\ \left| {z_{13} } \right| \le w_{xd} /2 \hfill \\ \end{gathered} \right. \Rightarrow \left\{ \begin{gathered} k_{qdz} = 0 \hfill \\ k_{hdz} = 0 \hfill \\ \end{gathered} \right. $$
2. When a contact collision has occurred between the front guidance sliding boots and the side of the pin rail, and when no contact collision has occurred between the rear guidance sliding boots and the side of the pin rail:
  $$ \left\{ \begin{gathered} \left| {z_{12} } \right| > w_{xd} /2 \hfill \\ \left| {z_{13} } \right| \le w_{xd} /2 \hfill \\ \end{gathered} \right. \Rightarrow \left\{ \begin{gathered} k_{qdz} \ne 0 \hfill \\ k_{hdz} = 0 \hfill \\ \end{gathered} \right. $$
3. When no contact collision has occurred between the front guidance sliding boots and the side of the pin rail, and when a contact collision has occurred between the rear guidance sliding boots and the side of the pin rail:
  $$ \left\{ \begin{gathered} \left| {z_{12} } \right| \le w_{xd} /2 \hfill \\ \left| {z_{13} } \right| > w_{xd} /2 \hfill \\ \end{gathered} \right. \Rightarrow \left\{ \begin{gathered} k_{qdz} = 0 \hfill \\ k_{hdz} \ne 0 \hfill \\ \end{gathered} \right. $$
4. When a contact collision has occurred between both the front and rear guidance sliding boots and the side of the pin rail:
  $$ \left\{ \begin{gathered} \left| {z_{12} } \right| > w_{xd} /2 \hfill \\ \left| {z_{13} } \right| > w_{xd} /2 \hfill \\ \end{gathered} \right. \Rightarrow \left\{ \begin{gathered} k_{qdz} \ne 0 \hfill \\ k_{hdz} \ne 0 \hfill \\ \end{gathered} \right. $$
  Combining the four contact cases above, the front and rear guidance sliding boots have a lateral normal contact rigidity with the pin rail is:
  13 $$ \left\{ {\begin{array}{*{20}c} {k_{qdz} = \frac{{2E_{dx} D}}{{\sqrt \pi \left( {1 - D} \right)}}\psi^{{\left( {2 - D} \right)/2}} \left( {z_{12} - \frac{{w_{xd} }}{2}} \right)^{D/2} \left[ {\left( {z_{12} - \frac{{w_{xd} }}{2}} \right)^{{\left( {1 - D} \right)/2}} - \left( {\frac{{w_{\mu xd} }}{2}} \right)^{{\left( {1 - D} \right)/2}} } \right]} & {} & {\left| {z_{12} } \right| > \frac{{w_{xd} }}{2}} \\ {k_{qdz} = 0} & {} & {\left| {z_{12} } \right| \le \frac{{w_{xd} }}{2}} \\ \end{array} } \right. $$
  14 $$ \left\{ {\begin{array}{*{20}c} {k_{hdz} = \frac{{2E_{dx} D}}{{\sqrt \pi \left( {1 - D} \right)}}\psi^{{\left( {2 - D} \right)/2}} \left( {z_{13} - \frac{{w_{xd} }}{2}} \right)^{D/2} \left[ {\left( {z_{13} - \frac{{w_{xd} }}{2}} \right)^{{\left( {1 - D} \right)/2}} - \left( {\frac{{w_{\mu xd} }}{2}} \right)^{{\left( {1 - D} \right)/2}} } \right]} & {} & {\left| {z_{13} } \right| > \frac{{w_{xd} }}{2}} \\ {k_{hdz} = 0} & {} & {\left| {z_{13} } \right| \le \frac{{w_{xd} }}{2}} \\ \end{array} } \right. $$
  where w_*μxd*_ is the critical contact cross-sectional area between the guidance sliding boots and the micro-convex body on the side of the pin rail for elastic- and plastic deformation.

### Shearer body and haulage unit connection rigidity model

According to the structure of the coal shearer, the rigidity model of the four rods can be used to describe the connection state between the body and the haulage unit. The relevant parameters of the hydraulic rods are shown in Table 2.

**Table 2:** Parameters related to hydraulic rods.

Designation of rod	Position of rod	Maximum rod travel/mm	Rod travel for pretension/mm
*l*_*ckx*_	Lower goaf	3460	6
*l*_*cks*_	Upper goaf	4370	7
*l*_*cmx*_	Lower coalface	3460	6
*l*_*cms*_	Upper coalface	6080	10

The equivalent connection rigidity of the coal shearer body to the haulage unit is:

15 $$ k_{cms} = \left\{ {\begin{array}{*{20}c} {\frac{{3E_{e} I_{e} }}{{l_{cms}^{3} }}} & {} & {z_{3} + z_{7} + \frac{j}{2} \cdot \left( {\eta_{z3} + \eta_{z7} } \right) - z_{6} \ne 10} \\ 0 & {} & {z_{3} + z_{7} + \frac{j}{2} \cdot \left( {\eta_{z3} + \eta_{z7} } \right) - z_{6} = 10} \\ \end{array} } \right. $$

16 $$ k_{cmx} = \left\{ {\begin{array}{*{20}c} {\frac{{3E_{e} I_{e} }}{{l_{cmx}^{3} }}} & {} & {z_{3} + z_{7} + \frac{j}{2} \cdot \left( {\eta_{z3} + \eta_{z7} } \right) - z_{6} \ne 6} \\ 0 & {} & {z_{3} + z_{7} + \frac{j}{2} \cdot \left( {\eta_{z3} + \eta_{z7} } \right) - z_{6} = 6} \\ \end{array} } \right. $$

17 $$ k_{cks} = \left\{ {\begin{array}{*{20}c} {\frac{{3E_{e} I_{e} }}{{l_{cks}^{3} }}} & {} & {z_{3} + z_{7} + \frac{j}{2} \cdot \left( {\eta_{z3} + \eta_{z7} } \right) - z_{6} \ne 7} \\ 0 & {} & {z_{3} + z_{7} + \frac{j}{2} \cdot \left( {\eta_{z3} + \eta_{z7} } \right) - z_{6} = 7} \\ \end{array} } \right. $$

18 $$ k_{ckx} = \left\{ {\begin{array}{*{20}c} {\frac{{3E_{e} I_{e} }}{{l_{ckx}^{3} }}} & {} & {z_{3} + z_{7} + \frac{j}{2} \cdot \left( {\eta_{z3} + \eta_{z7} } \right) - z_{6} \ne 6} \\ 0 & {} & {z_{3} + z_{7} + \frac{j}{2} \cdot \left( {\eta_{z3} + \eta_{z7} } \right) - z_{6} = 6} \\ \end{array} } \right. $$

### Ranging arm rigidity

The ranging arm rigidity model of coal shearer in the literature^25^, combined with the ranging arm own characteristics of coal shearer in this paper, the equivalent rigidity of the ranging arm is:

19 $$ k_{z2} = k_{z10} = \frac{{3E_{e} I_{e} }}{{p^{3} }} $$

where *E*_*e*_ is the modulus of elasticity of the ranging arm material of the coal shearer; *I*_*e*_ is the moment of inertia of the ranging arm section of the coal shearer.

## Drum load

### Drum load equation correction

Determination of the drum load, which is a prerequisite for the analysis of the dynamics of the whole machine. Due to the large error between the solution of the conventional drum load equation and the experimentally obtained load, as well as the experimentally obtained load as an external excitation, the system tends to diverge. In order to improve the accuracy of solving the dynamical characteristics of coal shearer under slant-cutting conditions, the traditional drum load equation^26^ is modified using experimental methods to propose a drum load with a traction speed correction factor of:

20 $$ \left\{ \begin{aligned} & R^{\prime}_{gx} = k_{vx} \cdot \sum\limits_{i = 1}^{{N_{c} }} {\left( {Z_{i} \cos \phi_{i} + Y_{i} \sin \phi_{i} } \right)} \hfill \\ & R^{\prime}_{gy} = k_{vy} \cdot \sum\limits_{i = 1}^{{N_{c} }} {\left( { - Z_{i} \sin \phi_{i} + Y_{i} \cos \phi_{i} } \right)} \hfill \\ & R^{\prime}_{gz} = k_{vz} \cdot \sum\limits_{i = 1}^{{N_{c} }} {\left( {X_{i} } \right)} \hfill \\ \end{aligned} \right. $$

where *R*_*gx*_ is the drum cutting loads in the traction direction; *R*_*gy*_ is the drum cutting loads in the vertical direction; *R*_*gz*_ is the drum cutting loads in the axial direction; *M*_*g*_ is the drum cutting torque; *N*_*c*_ is the number of drum picks involved in the cutting; *R*_*g*_ is the radius of the drum; *φ*_*i*_ is the angle between the i-th picks and the vertical direction of the drum; *X*_*i*_ is the lateral resistance of the i-th picks on the drum; *Y*_*i*_ is the traction resistance of the i-th picks on the drum; *Z*_*i*_ is the cutting resistance of the i-th picks on the drum; *k*_*vx*_, *k*_*vy*_ and *k*_*vz*_ are traction speed correction factors.

### Test program

**Figure 6:**
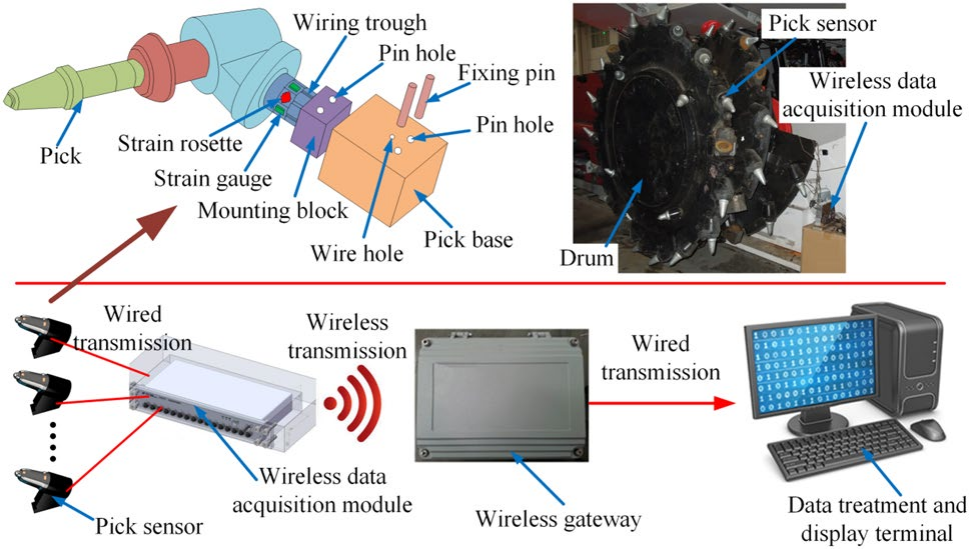
Pick load data acquisition system.

Using the mechanical testing and analysis experimental platform of the National Energy Extraction Equipment Research and Development Center's comprehensive mining operation, coal shearer cutting experiments are conducted and pick load data is collected. A total of nine pick sensors are fitted to the drum in the experiment, as shown in Fig. 6. The wires leading from each strain gauge and strain flower in the pick sensors are connected to the wireless acquisition module mounted on the end of the spiral blade of the drum through the wire slots and the wire holes in the tooth holders. The pick sensor transmits the collected data in a wired manner to the wireless acquisition module, which in turn transmits the data to the data acquisition terminal via wireless communication via a wireless gateway. The noisy signals collected at the wireless data acquisition terminal are first processed by noise reduction, then the three-way force of the drum cutter is obtained and finally the three-way force of the coal shearer drum is calculated.

In order to determine the traction speed correction factor in Eq. (20), the three-way drum cutting loads at traction speeds of 1.5 m/min, 2 m/min, 2.5 m/min, 3 m/min and 3.5 m/min is collected and calculated, and the average value of the three-way drum cutting loads at each traction speed is shown in Table 3 where the conventional calculated values are obtained from the conventional drum load equation, the experimental values are obtained from the drum load experiment.

**Table 3:** Experimental results and conventionally calculated values.

Operating conditions *v* (m/min)	Traction direction *R*_*gx*_/kN			Vertical direction *R*_*gy*_/kN			Axial direction *R*_*gz*_/kN		
	Testing value results	Traditional calculated value	Ratio of testing value to theoretical value	Testing value results	Traditional calculated value	Ratio of testing value to theoretical value	Testing value results	Traditional calculated value	Ratio of testing value to theoretical value
1.5	33.52	35.43	0.946	20.13	22.51	0.894	7.87	8.84	0.890
2	35.23	36.75	0.958	22.56	24.68	0.914	9.42	10.26	0.918
2.5	41.34	42.52	0.972	30.17	32.82	0.919	17.22	18.78	0.922
3	46.62	47.38	0.978	33.35	36.12	0.923	20.15	21.69	0.929
3.5	51.42	52.45	0.980	40.35	43.18	0.934	27.35	29.28	0.934

Traction speed correction factor:

21 $$ \left\{ \begin{gathered} k_{vx} = 0.932 + 0.018 \cdot v \hfill \\ k_{vy} = 0.872 + 0.018 \cdot v \hfill \\ k_{vz} = 0.869 + 0.020 \cdot v \hfill \\ \end{gathered} \right. $$

where *v* is the traction speed of the coal shearer.

The corrected values of the three-way load on the drum at a coal shearer traction speed of 3 m/min are selected and the conventional calculated values (average values) are compared with the experimental values (average values) as shown in Table 4. The corrected values for the three-way load of the drum are closer to the experimental values, with relative errors of 0.77%, 2.67% and 1.59% for the corrected and experimental values of the three-way load respectively.

**Table 4:** Comparison verification (average values).

Testing conditions	Traction direction *R*_*gx*_/kN			Vertical direction *R*_*gy*_/kN			Axial direction *R*_*gz*_/kN		
	Testing value results	Correction value	Error %	Testing value results	Correction value	Error %	Testing value results	Correction value	Error %
*v* = 3 m/min	46.62	46.98	0.77	33.35	34.23	2.67	20.15	20.47	1.59

## Analysis of simulation results

The Newmark-*β* method is used to solve the coupled vertical-pitch dynamics model for coal shearer:

1. Determine the mass matrix ***M***, the rigidity matrix ***K*** and the resistances matrix ***C.***
2. Given initial values ${\varvec{Y}}_{0}$ = 0, ${\dot{\varvec{Y}}}_{0}$ = 0, ${\varvec{\ddot{Y}}}_{0}$ = 0.
3. Set Δ*t* = 0.01, *δ* = 6/5, *β* = 1/12.
  $$a_{0} = \frac{1}{{\beta \Delta t^{2} }}$$; $$a_{1} = \frac{\delta }{\beta \Delta t}$$; $$a_{2} = \frac{1}{\beta \Delta t}$$; $$a_{3} = \frac{1}{2\beta } - 1$$; $$a_{4} = \frac{\delta }{\beta } - 1$$; $$a_{5} = \frac{\delta }{2\beta } - 1$$; $$a_{6} = \Delta t\left( {1 - \delta } \right)$$; $$a_{7} = \delta \Delta t$$.
4. $${\hat{\varvec{K}}} = a_{0} {\varvec{M}} + a_{1} {\varvec{C}} + {\varvec{K}}$$; $${\hat{\varvec{K}}\varvec{X}}_{t + \Delta t} = {\hat{\varvec{F}}}_{t + \Delta t}$$; $${\hat{\varvec{K}}} = {\varvec{LDL}}^{T}$$.
5. $${\hat{\varvec{F}}}_{t + \Delta t} = {\varvec{F}}_{t + \Delta t} + {\varvec{\rm M}}\left( {a_{0} {\varvec{Y}}_{t} + a_{2} {\dot{\varvec{Y}}}_{t} + a_{3} {\varvec{\ddot{Y}}}_{t} } \right) + C\left( {a_{1} {\varvec{Y}}_{t} + a_{4} {\dot{\varvec{Y}}}_{t} + a_{5} {\varvec{\ddot{Y}}}_{t} } \right)$$.
6. $${\varvec{LDL}}^{T} {\varvec{Y}}_{t + \Delta t} = {\hat{\varvec{F}}}_{t + \Delta t} \Rightarrow {\varvec{Y}}_{t + \Delta t}$$.
7. $${\varvec{\ddot{Y}}}_{t + \Delta t} = a_{0} \left( {{\varvec{Y}}_{t + \Delta t} - {\varvec{Y}}_{t} } \right) - a_{2} {\dot{\varvec{Y}}}_{t} - a_{3} {\varvec{\ddot{Y}}}_{t}$$; $${\dot{\varvec{Y}}}_{t + \Delta t} = {\dot{\varvec{Y}}}_{t} + a_{6} {\varvec{\ddot{Y}}}_{t} + a_{7} {\varvec{\ddot{Y}}}_{t + \Delta t}$$.

According to the best economic and efficient working parameters obtained from the long-term work of coal shearer in coal mine tunnels, the pitch angle is set to 0 and the lifting angle of the front swing arm is 27° in the solution process. Using the drum load at *v* = 3 m/min and *f* = 3 as the external excitation, the vibration response curve of each key component of the coal shearer is intercepted over a period of 20 s.

### Vibration displacement and swing

**Figure 7:**
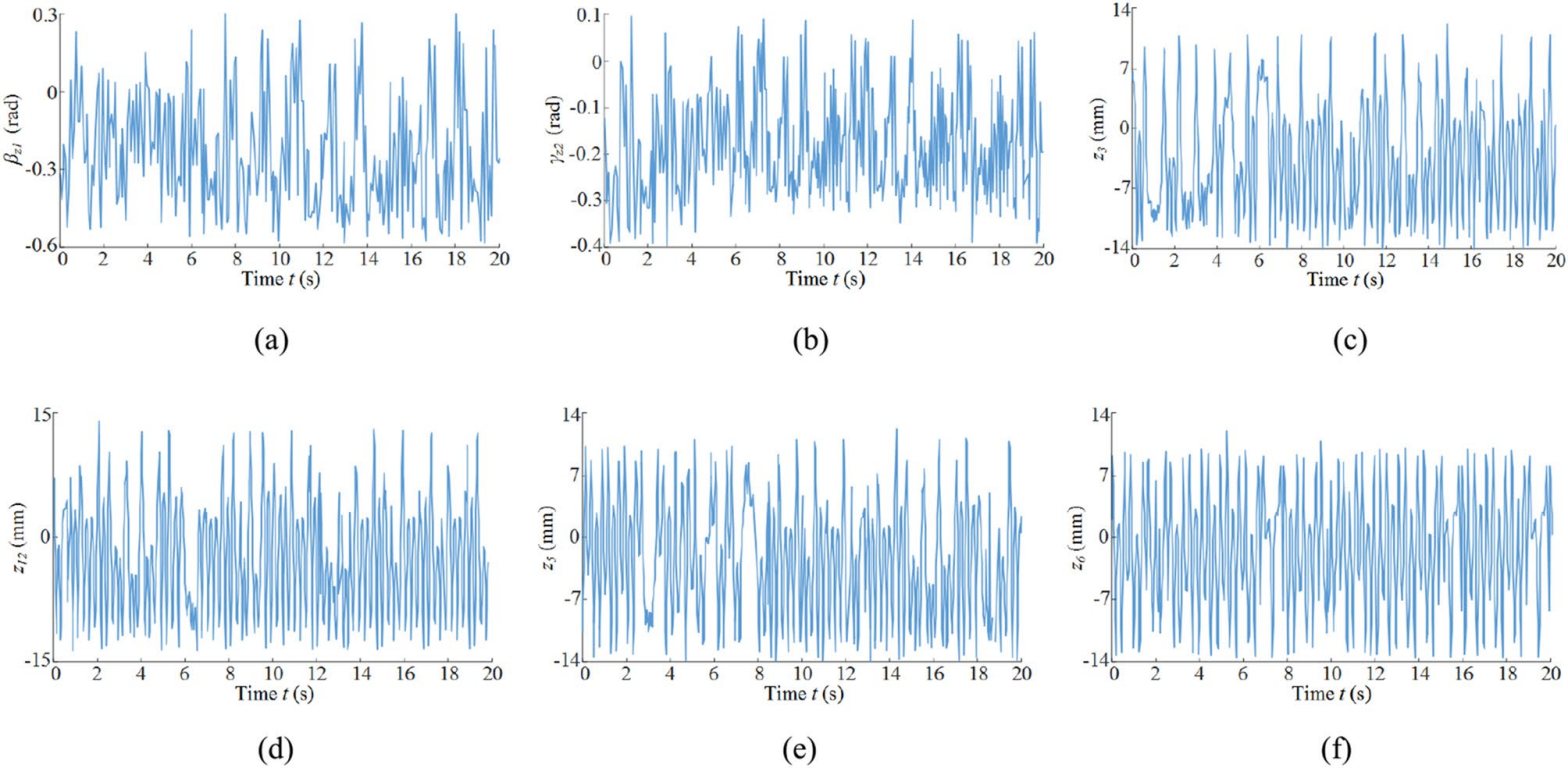
Vibration displacement and swing curve: (**a**) vibration-induced swing for front drum, (**b**) vibration-induced swing for front ranging arm, (**c**) vibration-induced displacement for front traction unit, (**d**) vibration-induced displacement for front walking unit, (**e**) vibration-induced displacement for front support unit, (**f**) vibration-induced displacement for body.

Figure 7 shows the vibration swing and vibration displacement of each key component under the slant-cutting condition, the vibration displacement of each key component of the coal shearer under the slant-cutting condition is negative for most of the time, and the vibration displacement direction is away from the coal wall. Due to the front drum being the external excitation input and the assumption of the front end of the ranging arm as a massless flexible beam, the front drum has the largest vibration swing angle and the largest vibration swing angle fluctuation range, which fluctuates from − 0.6 to 0.3 rad. Also influenced by the vibration of the front drum and the characteristics of the connection with the haulage unit, as well as the fact that the mass of the haulage unit is much larger than that of the ranging arm, the vibration swing angle of the front ranging arm is smaller compared to that of the front drum, and the fluctuation range is smaller. The vibration swing angle fluctuation range is − 0.4 rad to 0.1 rad. The front ranging arm is also influenced by the rigidity characteristics of the ranging arm itself, and most of the time the front ranging arm vibrates at the opposite angle to the front drum. Due to the large mass of the front haulage unit, front walking unit, front support unit and shearer body, the fluctuation range of vibration displacement is basically stable, where the front haulage unit, front walking unit and front support unit are rigidly connected, their fluctuation range of vibration displacement is basically the same, the fluctuation range of vibration displacement of the front haulage unit and support unit is – 14–11 mm. The vibration displacement of the front walking unit fluctuates in the range of − 15 to 15 mm, due to the influence of the connection gap between the guidance sliding boots and the pin rail.

### Vibration acceleration

**Figure 8:**
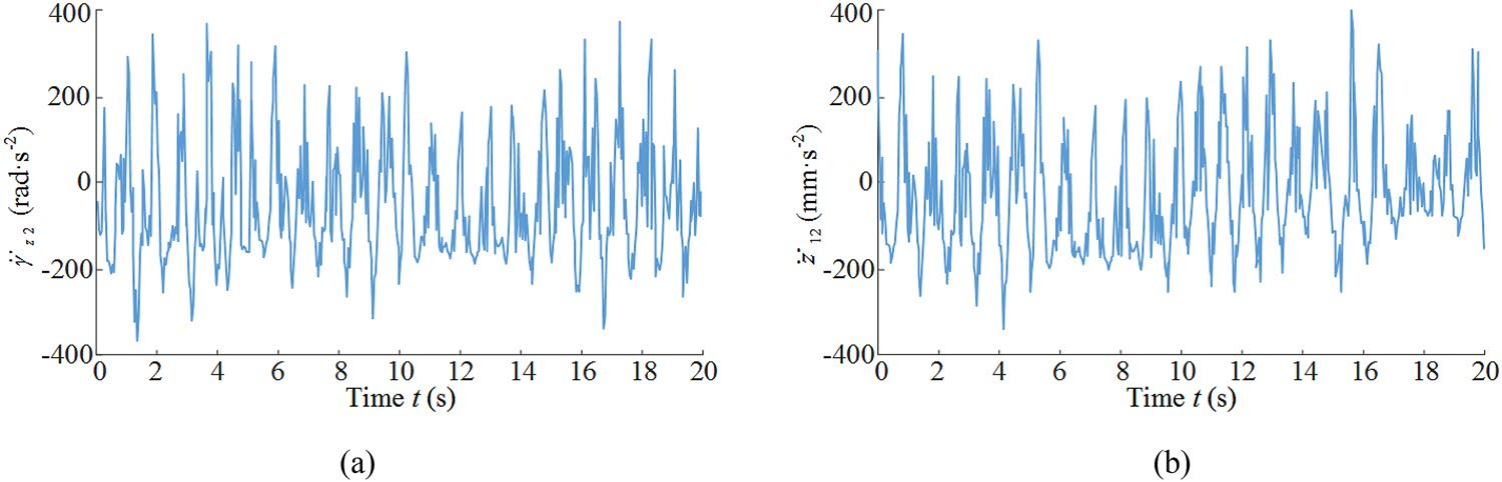
Vibration acceleration curve: (**a**) vibration-induced acceleration for front ranging arm, (**b**) vibration-induced acceleration for front walking unit.

In the actual structure of the ranging arm and walking unit contains a long distance multi-stage composite gearing system, and from the vibration displacement analysis can be seen, the vibration characteristics of the front ranging arm and front walking unit has included the vibration characteristics of other key components, with a certain degree of representation, so the following vibration characteristics of the front ranging arm and front walking unit focus on the analysis. Figure 8a shows the vibration acceleration curve for front ranging arm. Due to the front drum as input to the system load, the vibration excitation generated directly affects the front ranging arm, resulting in a large load impact on the front ranging arm, and the influence of the connection gap with the front haulage unit, the ranging arm and the haulage unit produce a strong impact, resulting in a large peak of the vibration acceleration curve of the front ranging arm at some moments. The front ranging arm's vibration acceleration curve has a large peak at some moments, but then returns to stability, with a stable fluctuation range of − 300 rad/s^2^ to 300 rad/s^2^. Figure 8b is the vibration acceleration curve of front walking unit, with the load in the transmission process gradually decay, the vibration acceleration curve of front walking unit fluctuation range and the front ranging arm is relatively small, by the guidance sliding boots and the pin rail with the influence of gaps, the guidance sliding boots and the pin rail produce strong impact, resulting in the vibration acceleration curve of front walking unit in some moments fluctuation of the peak is larger, and then restore stability, it's stable fluctuation range is – 200–200 mm/s^2^.

### Frequency domain response

**Figure 9:**
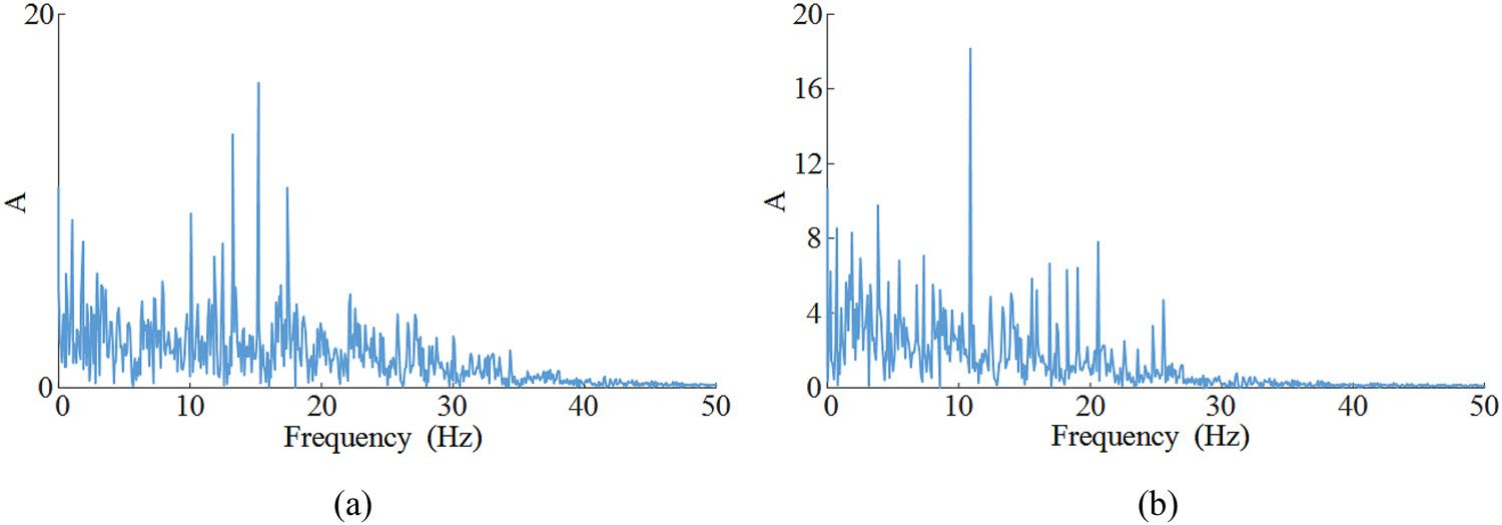
Vibration spectrum of key coal shearer components under slant-cutting tool conditions: (**a**) vibration spectrogram for front ranging arm, (**b**) vibration spectrogram for front walking unit.

Figure 9 shows the frequency domain response curves of the vibration of the front ranging arm and front walking unit, with main frequencies of 16.63 Hz and 12.14 Hz respectively, and with some lower frequency characteristics interspersed in the vibration spectrum.

### Analysis of dynamics under different operating parameters

**Figure 10:**
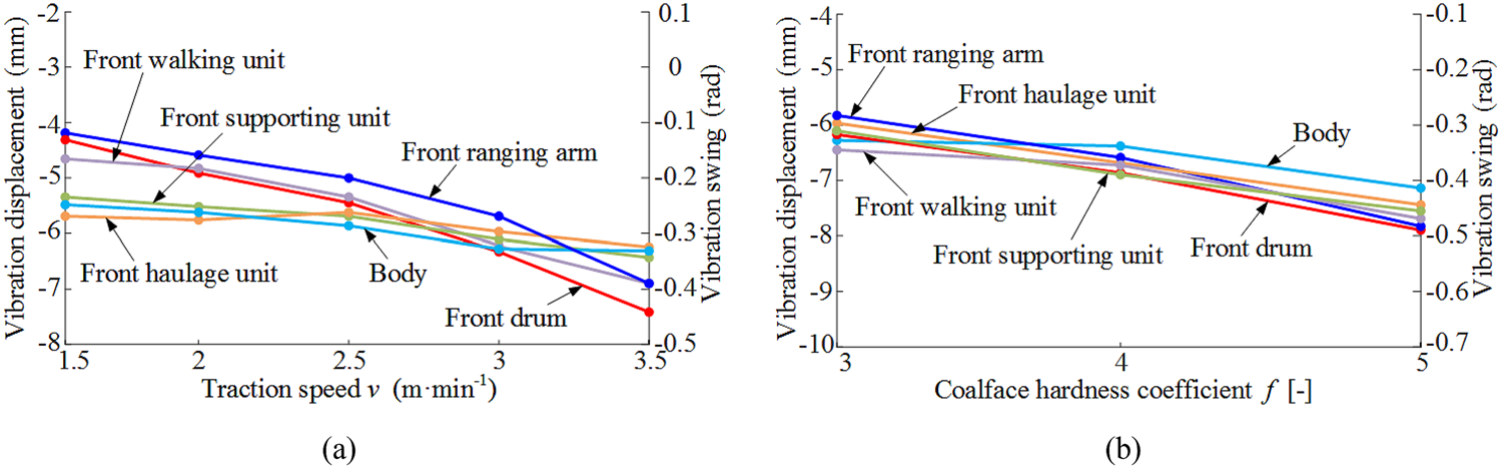
Vibration displacement of key coal shearer components under slant-cutting conditions with different operating parameters: (**a**) different traction speed, (**b**) different coalface hardness coefficient.

Using the single variable method, the vibration displacement characteristics of each part of the coal shearer under different traction speeds and different coalface hardness coefficient are solved, and the average values of the vibration displacement curves of each part are obtained, and the vibration displacement curves of each part of the coal shearer under slant-cutting conditions with different operating parameters are plotted in Fig. 10.

When *f* = 3, *v* = 1.5–3.5 m/min, as shown in Fig. 10a, the change of traction speed on the front drum, front ranging arm and front walking unit vibration swing and displacement influence more, with the increase of traction speed of the three vibration swing and displacement increased, its change range are − 0.16 rad  to  − 0.44 rad, − 0.15 rad  to  − 0.39 rad and − 4.5 mm to − 6.9 mm respectively.

When *v* = 3 m/min and *f* = 3–5, as shown in Fig. 10b, with the increase of coalface hardness coefficient only the vibration swing of the front drum and front ranging arm produced small changes, as the coalface hardness coefficient gradually became larger, the average values of vibration swing angle of the front ranging arm and front drum are gradually close to each other, the vibration swing of the two varied in the ranges of − 0.32 rad to − 0.49 rad, − 0.28 rad to − 0.48 rad.

## Experimental test

### Experimental platform

**Figure 11:**
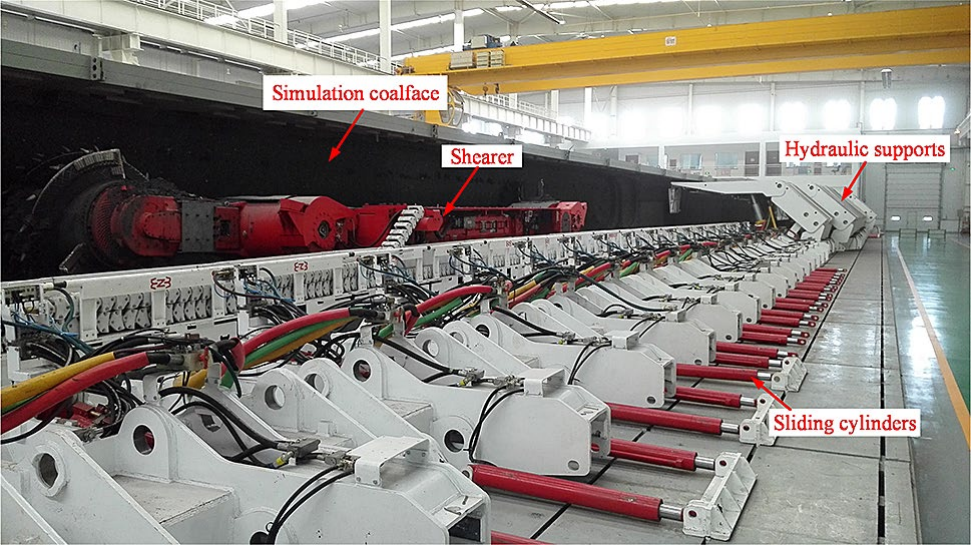
Experimental platform for mechanical testing and analysis of the comprehensive mining operation.

Using the mechanical testing and analysis platform of the National Energy Extraction Equipment Research and Development Experiment Center, experiments on cutting and mechanical testing of coal shearer under slant-cutting conditions are carried out to verify the solution results. As shown in Fig. 11, the experimental platform mainly consists of a 1:1 simulated coal wall, coal shearer, scraper conveyor, hydraulic support, sliding cylinder and data acquisition system, where the coal shearer model is MG500/1130-WD and the scraper conveyor model is SGZ1000/1050.

In order to ensure that the data measured in the experiment is realistic and reliable, the simulated coal wall should simulate the geological structure and mechanical properties of the real working face coal seam as far as possible. As the largest supply base of high quality power coal in China, the coal seams in Datong, Shanxi, have better joint development, less impurities, high heat generation and high hardness, and are representative of coal seams in various regions of China, so the coal seams in Datong, Shanxi, are chosen as the simulated object for the experimental coal wall. The simulated coal wall is mainly made of coal, supplemented by raw materials such as cement. The selected coal and cement are mixed by water and an appropriate amount of water reducer is added, and the model coal wall is poured in the form of layer by layer to ensure that the poured simulated coal wall had the characteristics of bedding and jointing, each layer being 300 mm, where the main raw material parameters are shown in Table 5. The simulated coal wall is 70 m long, 4 m wide and 1.5 m high, with a coalface hardness coefficient of *f* = 3 for the first 35 m and *f* = 4 for the last 35 m.

**Table 5:** Main raw material parameters for simulated coal walls.

Coal (particle size)		Ordinary silicate cement	
Fine aggregates	Coarse aggregates	Strength grade	Extra coefficient
≤ 5 mm	5–50 mm	32.5 MPa	1.05 [–]

### Data acquisition and transmission system

**Figure 12:**
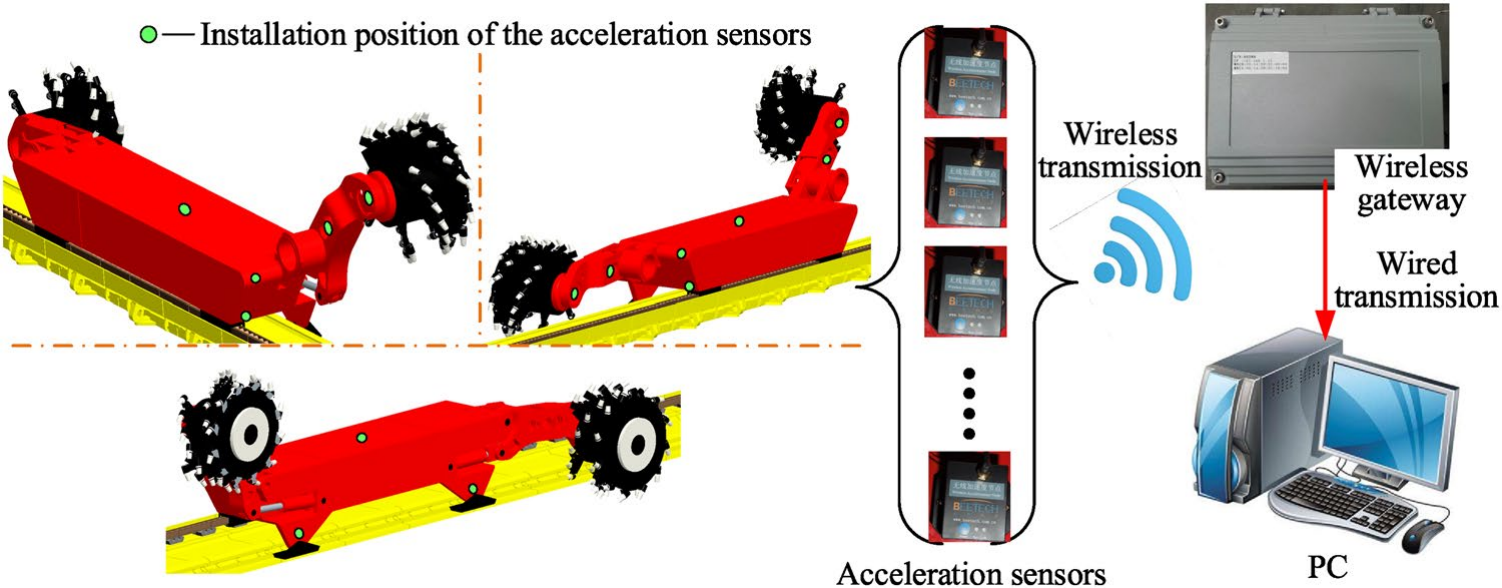
Vibration acceleration data acquisition system for key coal shearer components.

As shown in Fig. 12, the A301 wireless acceleration sensor, which is a combination of power supply module, signal acquisition and processing module, and wireless transceiver module, is developed by Beijing Beetech Technology Co. The sensor uploads the collected data to the wireless gateway by means of wireless transmission. The wireless gateway transmits the data to the terminal PC by means of wired transmission, and finally the collected data is processed on the PC and displayed in the form of graphics.

**Figure 13:**
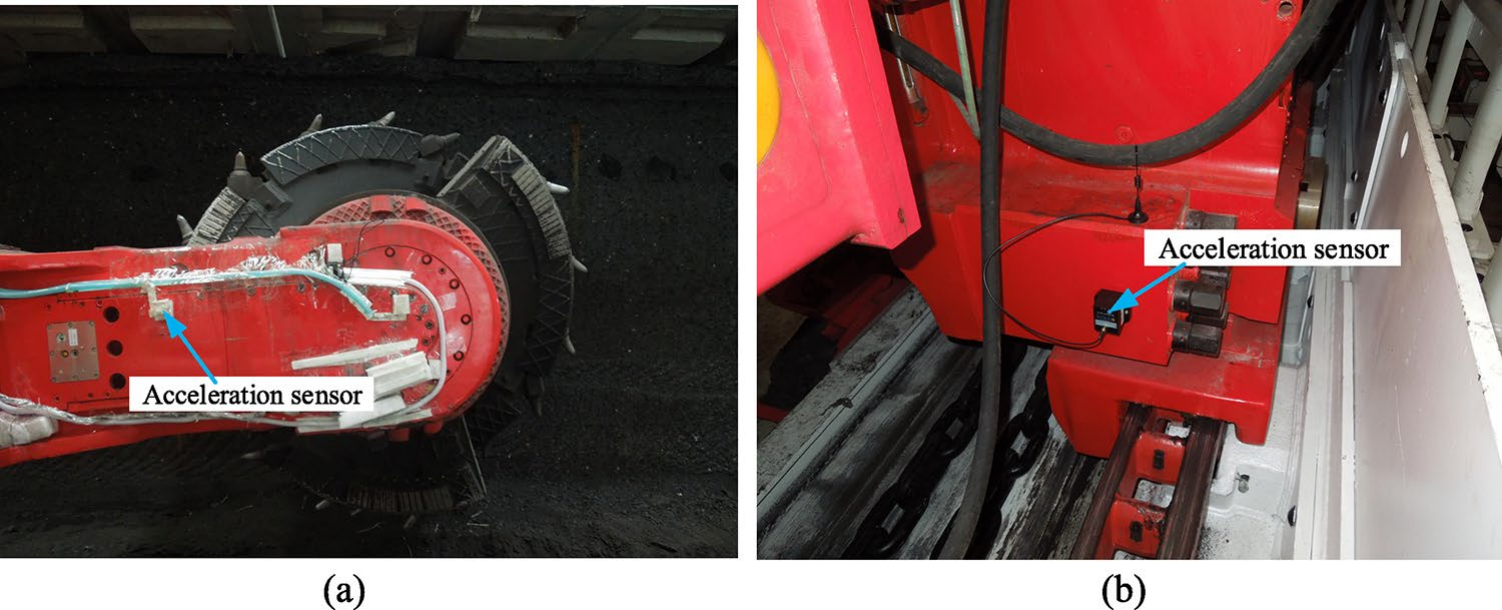
Ranging arm and walking unit vibration acceleration sensor mounting position: (**a**) ranging arm sensor arrangement, (**b**) walking unit sensor arrangement.

Mainly introduces the mounting position of the ranging arm and walking unit acceleration sensors for coal shearer. As shown in Fig. 13a, the position of the ranging arm's center of gravity is chosen to mount the acceleration sensor and the sensor is suitably encapsulated to take account of the lack of influence from falling coal. As shown in Fig. 13b, the acceleration sensor is selected to be mounted at the end of the walking unit and close to the center of gravity, taking into account that the sensor is not affected by the coal fall, the scraper conveyor baffle and the pin rail.

### Comparative theoretical and experimental analysis

**Figure 14:**
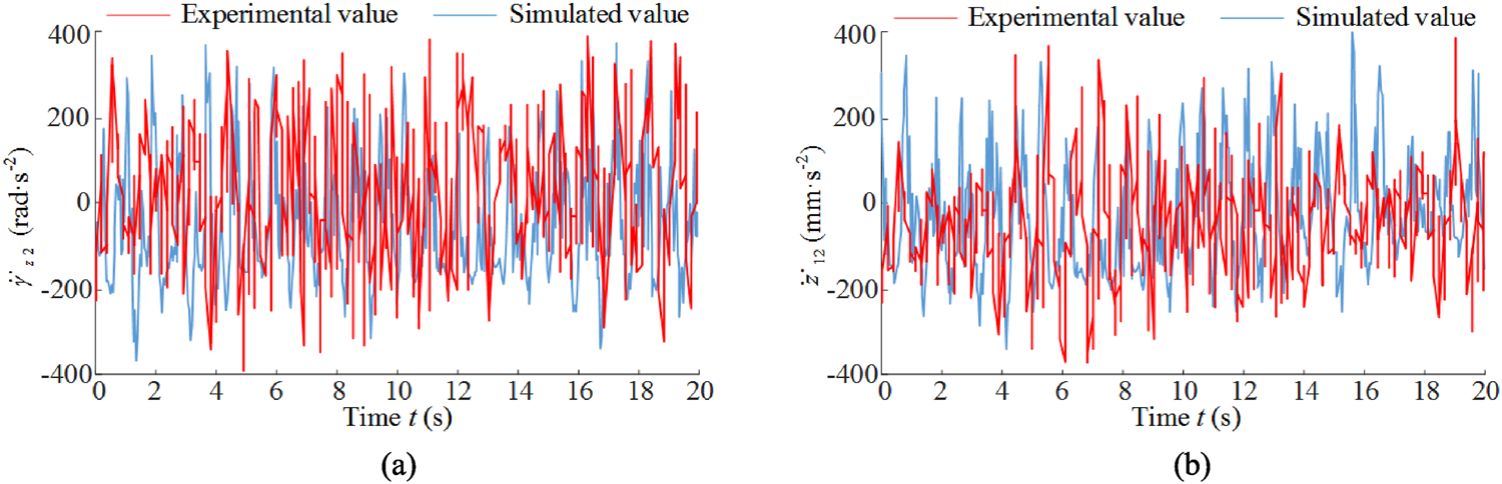
Vibration acceleration: (**a**) ranging arm, (**b**) walking unit.

**Figure 15:**
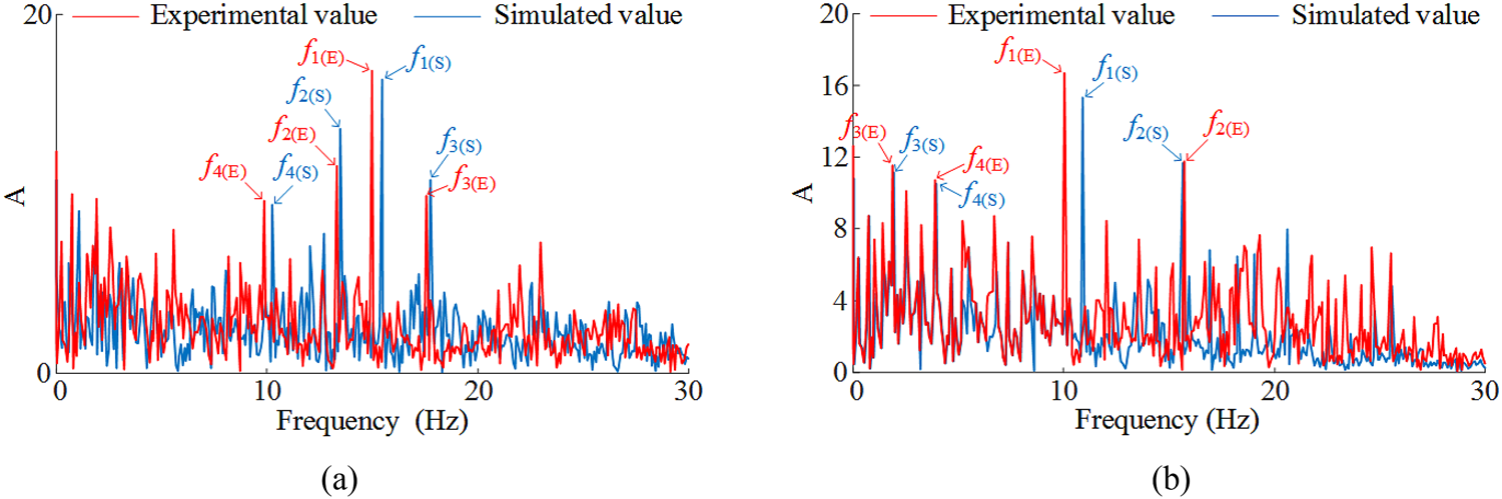
Vibration frequency spectrum: (**a**) ranging arm, (**b**) walking unit.

The vibration acceleration curves and frequency domain response curves of the front ranging arm and front walking unit obtained by solving for the parameters *v* = 3 m/min and *f* = 3 are compared and analyzed with the experimental values, Fig. 14 shows the vibration acceleration curves and Fig. 15 shows the vibration frequency spectrum. It can be seen that the simulated values (average values) and experimental values (average values) of the vibration acceleration curves of the two are basically the same in terms of fluctuation frequency and fluctuation range; the simulated and experimental values of the main vibration frequencies of the two are less different and the distribution of the lower frequency characteristics is basically the same. As shown in Tables 6 and 7, the simulated values are compared with the laboratory and the differences between the simulated and laboratory values are small, with errors of less than 10%.

**Table 6:** Comparison between simulated values (average values) and experimental values (average values) of vibration displacement.

	Ranging arm	Walking unit
Simulation value	− 0.217 rad/s^2^	− 0.154 mm/s^2^
Test results	− 0.198 rad/s^2^	− 0.171 mm/s^2^
Relative error	9.60%	9.94%

**Table 7:** Comparison between simulated and experimental values of vibration frequency.

	Ranging arm (Hz)				Walking unit (Hz)			
	*f*_1_	*f*_2_	*f*_3_	*f*_4_	*f*_1_	*f*_2_	*f*_3_	*f*_4_
Simulation value	16.63	13.11	17.84	10.53	12.14	15.19	1.55	2.36
Test results	15.91	12.82	17.33	9.78	11.09	15.27	1.59	2.43
Relative error	4.53%	2.26%	2.94%	7.67%	9.47%	0.52%	2.51%	2.88%

Error analysis: (1) In the theoretical analysis, the influence of the vibration of the coal shearer's cutting gear drive system and the walking unit gear drive system on the vibration of the whole machine is not considered; (2) In the theoretical analysis, the influence of the gangue under the sliding shoe of the coal shearer on the vibration of the whole machine is not considered; (3) In the theoretical analysis, the influence of the vibration of the electrical and hydraulic systems of the coal shearer on the vibration of the whole machine is not considered; (4) In the theoretical analysis, the design dimensions of the coal shearer are used for analysis, not the actual dimensions of the coal shearer, and the influence of assembly errors on the design dimensions of the whole coal shearer in the assembly process is not considered; (5) There are errors in the process of experimentation and data processing.

## Conclusion

The contact characteristics between the key components of the coal shearer, as well as the support characteristics, are taken into account, and the dynamic characteristics of the coal shearer under slant-cutting conditions are solved and analyzed, with the following results:

1. A model for the tangential rigidity of the slide shoes-middle groove, the normal rigidity of the ranging arm-haulage unit with gaps, and the multi-surface contact rigidity of the guidance sliding boots-pin rail with gaps are developed using three-dimensional fractal theory. A model for the rigidity of the front and rear haulage units to the body and a model for the rigidity of the ranging arm are developed using Hooke's law, and based on these models, a 13-degree-of-freedom non-linear dynamics model for the coal shearer under slant-cutting conditions is constructed using multi-body dynamics theory.
2. A drum correction load with a traction speed correction factor is proposed, the correction factor is determined by experimental methods, and the correction results are analyzed in comparison with conventional values and experiments. The analysis shows that the corrections have a much smaller error with the laboratory, with errors of 0.77%, 2.67% and 1.59% in the traction direction, vertical direction and axial direction respectively.
3. Using the drum correction load as the external excitation, the Newmark-*β* method is used to solve the dynamics model of the coal shearer, and the vibration response curves of each key component of the coal shearer under the slant-cutting working condition are obtained. A detailed analysis of the vibration characteristics of the front ranging arm and front walking unit is carried out for the operating parameters of *v* = 3 m/min and *f* = 3. Swing and displacement fluctuations of − 0.4 rad to 0.1 rad and − 15 to 15 mm for the front ranging arm and front drum respectively. Vibration accelerations of − 300 rad/s^2^ to 300 rad/s^2^ and − 200 mm/s^2^ to 200 mm/s^2^ respectively. Main vibration frequencies of 16.63 Hz and 12.14 Hz respectively. Finally, the single variable method is used to solve the vibration displacement characteristics of each key component of the coal shearer under different traction speed and coalface hardness coefficient, and the vibration swing and displacement of the front drum, front ranging arm and front walking unit are influenced by the change of traction speed. As the traction speed increases the vibration swing and displacement of the three increases, while the change in coalface hardness coefficient has less effect on the vibration displacement of the key components of the coal shearer.
4. The results are verified by experimental methods, and the vibration acceleration curves and frequency domain response curves of the front ranging arm and front walking unit obtained from the solutions are compared with the experimental values. As the influence of the internal structure of the coal shearer, the transmission system, the electrical system and the assembly process are not taken into account in the theoretical analysis, the solution results have some errors with the experimental measurements, but the relative errors are less than 10%, thus verifying the accuracy of the established dynamics model of the coal shearer under slant-cutting conditions.

The results of the study can guide the optimal control of coal shearer operating parameters under slant-cutting conditions, and can provide a theoretical basis for the stability of coal shearer and the fatigue life prediction of the key components.

## Appendix

The matrix ***M*** is:

$$ {\varvec{M}} = \left[ {\begin{array}{*{20}c} {{\varvec{M}}_{{\varvec{1}}} } & {} \\ {} & {{\varvec{M}}_{{\varvec{2}}}^{{}} } \\ \end{array} } \right] $$

$${\varvec{M}}_{{\varvec{1}}} = \left[ {\begin{array}{*{20}c} {m_{1} \cdot p^{2} } & {e \cdot m_{1} \cdot p} & {m_{1} \cdot p \cdot \sin \alpha_{z1} } \\ {e \cdot m_{1} \cdot p} & {e^{2} \cdot \left( {m_{1} + m_{2} } \right)} & {e \cdot \left( {m_{1} + m_{2} } \right) \cdot \sin \alpha_{z1} } \\ {m_{1} \cdot p \cdot \sin \alpha_{z1} } & {e \cdot \left( {m_{1} + m_{2} } \right) \cdot \sin \alpha_{z1} } & {m_{1} + m_{2} + m_{3} } \\ \end{array} } \right];$$

$${\varvec{M}}_{{\varvec{2}}} = \left[ {\begin{array}{*{20}c} {m_{7} + m_{10} + m_{11} } & 0 & 0 & 0 & 0 & 0 & 0 & 0 & {M_{4,12} } & {M_{4,13} } \\ 0 & {m_{5} } & 0 & 0 & 0 & 0 & 0 & 0 & 0 & 0 \\ 0 & 0 & {m_{6} } & 0 & 0 & 0 & 0 & 0 & 0 & 0 \\ 0 & 0 & 0 & {m_{8} } & 0 & 0 & 0 & 0 & 0 & 0 \\ 0 & 0 & 0 & 0 & {m_{12} } & 0 & 0 & 0 & 0 & 0 \\ 0 & 0 & 0 & 0 & 0 & {m_{13} } & 0 & 0 & 0 & 0 \\ 0 & 0 & 0 & 0 & 0 & 0 & {I_{z3} } & 0 & 0 & 0 \\ 0 & 0 & 0 & 0 & 0 & 0 & 0 & {I_{z7} } & 0 & 0 \\ {M_{124} } & 0 & 0 & 0 & 0 & 0 & 0 & 0 & {e^{2} \cdot \left( {m_{10} + m_{11} } \right)} & {M_{12,13} } \\ {M_{134} } & 0 & 0 & 0 & 0 & 0 & 0 & 0 & {M_{13,12} } & {m_{11} \cdot p^{2} } \\ \end{array} } \right];$$

$$M_{4,12} = e \cdot \left( {m_{10} + m_{11} } \right) \cdot \sin \alpha_{z11}$$; $$M_{4,13} = m_{11} \cdot p \cdot \sin \alpha_{z11}$$; $$M_{12,4} = e \cdot \left( {m_{10} + m_{11} } \right) \cdot \sin \alpha_{z11}$$;

$$M_{12,13} = e \cdot m_{11} \cdot p$$; $$M_{13,4} = m_{11} \cdot p \cdot \sin \alpha_{z11}$$; $$M_{13,12} = e \cdot m_{11} \cdot p$$。

The matrix ***C*** is:

$$ {\varvec{C}} = \left[ {\begin{array}{*{20}c} {{\varvec{C}}_{{\varvec{1}}} } & {{\varvec{C}}_{{\varvec{3}}} } \\ {{\varvec{C}}_{{\varvec{4}}} } & {{\varvec{C}}_{{\varvec{2}}} } \\ \end{array} } \right] $$

$${\varvec{C}}_{{\varvec{1}}} = \left[ {\begin{array}{*{20}c} 0 & 0 & 0 & 0 & 0 & 0 & 0 \\ 0 & {e^{2} \cdot c_{zz2} } & { - e \cdot c_{zz2} } & 0 & 0 & 0 & 0 \\ 0 & { - e \cdot c_{zz2} } & {C_{3,3} } & {C_{3,4} } & { - c_{zz5} } & {C_{3,6} } & 0 \\ 0 & 0 & {C_{4,3} } & {C_{4,4} } & 0 & {C_{4,6} } & { - c_{zz8} } \\ 0 & 0 & { - c_{zz5} } & 0 & {c_{qpz} + c_{zz5} } & 0 & 0 \\ 0 & 0 & {C_{6,3} } & {C_{6,4} } & 0 & {C_{6,6} } & 0 \\ 0 & 0 & 0 & { - c_{zz8} } & 0 & 0 & {c_{hpz} + c_{zz8} } \\ \end{array} } \right];$$ $${\varvec{C}}_{{\varvec{2}}} = \left[ {\begin{array}{*{20}c} {C_{8,8} } & 0 & 0 & 0 & 0 & 0 \\ 0 & {C_{9,9} } & 0 & 0 & 0 & 0 \\ 0 & 0 & {C_{10,10} } & {C_{10,11} } & 0 & 0 \\ 0 & 0 & {C_{11,10} } & {C_{11,11} } & { - \left( {e \cdot r \cdot c_{zz10} } \right)/2} & 0 \\ 0 & 0 & 0 & { - \left( {e \cdot r \cdot c_{zz10} } \right)/2} & {e^{2} \cdot c_{zz10} } & 0 \\ 0 & 0 & 0 & 0 & 0 & 0 \\ \end{array} } \right];$$

$${\varvec{C}}_{{\varvec{3}}} = \left[ {\begin{array}{*{20}c} 0 & 0 & 0 & 0 & 0 & 0 \\ 0 & 0 & { - \left( {e \cdot r \cdot c_{zz2} } \right)/2} & 0 & 0 & 0 \\ { - c_{zz12} } & 0 & {C_{3,10} } & {C_{3,11} } & 0 & 0 \\ 0 & { - c_{zz13} } & {C_{4,10} } & {C_{4,11} } & { - e \cdot c_{zz10} } & 0 \\ 0 & 0 & 0 & 0 & 0 & 0 \\ 0 & 0 & {C_{6,10} } & {C_{6,11} } & 0 & 0 \\ 0 & 0 & 0 & 0 & 0 & 0 \\ \end{array} } \right];$$ $${\varvec{C}}_{{\varvec{4}}} = \left[ {\begin{array}{*{20}c} 0 & 0 & { - c_{zz12} } & 0 & 0 & 0 & 0 \\ 0 & 0 & 0 & { - c_{zz13} } & 0 & 0 & 0 \\ 0 & { - \left( {e \cdot r \cdot c_{zz2} } \right)/2} & {C_{10,3} } & {C_{10,4} } & 0 & {C_{10,6} } & 0 \\ 0 & 0 & {C_{11,3} } & {C_{11,4} } & 0 & {C_{11,6} } & 0 \\ 0 & 0 & 0 & { - e \cdot c_{zz10} } & 0 & 0 & 0 \\ 0 & 0 & 0 & 0 & 0 & 0 & 0 \\ \end{array} } \right];$$

$$C_{3,3} = c_{cks} + c_{ckx} + c_{cms} + c_{cmx} + c_{zz2} + c_{zz5} + c_{zz12};$$ $$C_{3,4} = c_{cks} + c_{ckx} + c_{cms} + c_{cmx};$$ $$C_{3,6} = - \left( {c_{cks} + c_{ckx} + c_{cms} + c_{cmx} } \right);$$

$$C_{3,10} = \left( {l_{cks} \cdot c_{cks} + l_{ckx} \cdot c_{ckx} + l_{cms} \cdot c_{cms} + l_{cmx} \cdot c_{cmx} + r \cdot c_{zz2} } \right)/2;$$ $$C_{3,11} = \left( {l_{cks} \cdot c_{cks} + l_{ckx} \cdot c_{ckx} + l_{cms} \cdot c_{cms} + l_{cmx} \cdot c_{cmx} } \right)/2;$$

$$C_{4,3} = c_{cks} + c_{ckx} + c_{cms} + c_{cmx};$$ $$C_{4,4} = c_{cks} + c_{ckx} + c_{cms} + c_{cmx} + c_{zz8} + c_{zz10} + c_{zz13};$$ $$C_{4,6} = - \left( {c_{cks} + c_{ckx} + c_{cms} + c_{cmx} } \right);$$

$$C_{4,10} = \left( {l_{cks} \cdot c_{cks} + l_{ckx} \cdot c_{ckx} + l_{cms} \cdot c_{cms} + l_{cmx} \cdot c_{cmx} } \right)/2;$$ $$C_{4,11} = \left( {l_{cks} \cdot c_{cks} + l_{ckx} \cdot c_{ckx} + l_{cms} \cdot c_{cms} + l_{cmx} \cdot c_{cmx} + r \cdot c_{zz10} } \right)/2;$$

$$C_{6,3} = - \left( {c_{cks} + c_{ckx} + c_{cms} + c_{cmx} } \right);$$ $$C_{6,4} = - \left( {c_{cks} + c_{ckx} + c_{cms} + c_{cmx} } \right);$$ $$C_{6,6} = c_{cks} + c_{ckx} + c_{cms} + c_{cmx};$$

$$C_{6,10} = - \left( {l_{cks} \cdot c_{cks} + l_{ckx} \cdot c_{ckx} + l_{cms} \cdot c_{cms} + l_{cmx} \cdot c_{cmx} } \right)/2;$$ $$C_{6,11} = - \left( {l_{cks} \cdot c_{cks} + l_{ckx} \cdot c_{ckx} + l_{cms} \cdot c_{cms} + l_{cmx} \cdot c_{cmx} } \right)/2$$

$$C_{8,8} = c_{qdm} + c_{qdz} + c_{zz12};$$ $$C_{9,9} = c_{hdm} + c_{hdz} + c_{zz13};$$ $$C_{10,3} = \left( {l_{cks} \cdot c_{cks} + l_{ckx} \cdot c_{ckx} + l_{cms} \cdot c_{cms} + l_{cmx} \cdot c_{cmx} + r \cdot c_{zz2} } \right)/2;$$

$$C_{10,4} = \left( {l_{cks} \cdot c_{cks} + l_{ckx} \cdot c_{ckx} + l_{cms} \cdot c_{cms} + l_{cmx} \cdot c_{cmx} } \right)/2;$$ $$C_{10,6} = - \left( {l_{cks} \cdot c_{cks} + l_{ckx} \cdot c_{ckx} + l_{cms} \cdot c_{cms} + l_{cmx} \cdot c_{cmx} } \right)/2;$$

$$C_{10,10} = \left( {l_{cks}^{2} \cdot c_{cks} + l_{ckx}^{2} \cdot c_{ckx} + l_{cms}^{2} \cdot c_{cms} + l_{cmx}^{2} \cdot c_{cmx} + r^{2} \cdot c_{zz2} } \right)/4;$$ $$C_{10,11} = \left( {l_{cks}^{2} \cdot c_{cks} + l_{ckx}^{2} \cdot c_{ckx} + l_{cms}^{2} \cdot c_{cms} + l_{cmx}^{2} \cdot c_{cmx} } \right)/4;$$

$$C_{11,3} = \left( {l_{cks} \cdot c_{cks} + l_{ckx} \cdot c_{ckx} + l_{cms} \cdot c_{cms} + l_{cmx} \cdot c_{cmx} } \right)/2;$$ $$C_{11,4} = \left( {l_{cks} \cdot c_{cks} + l_{ckx} \cdot c_{ckx} + l_{cms} \cdot c_{cms} + l_{cmx} \cdot c_{cmx} + r \cdot c_{zz10} } \right)/2;$$

$$C_{11,6} = - \left( {l_{cks} \cdot c_{cks} + l_{ckx} \cdot c_{ckx} + l_{cms} \cdot c_{cms} + l_{cmx} \cdot c_{cmx} } \right)/2;$$ $$C_{11,10} = \left( {l_{cks}^{2} \cdot c_{cks} + l_{ckx}^{2} \cdot c_{ckx} + l_{cms}^{2} \cdot c_{cms} + l_{cmx}^{2} \cdot c_{cmx} } \right)/4;$$

$$C_{11,11} = \left( {l_{cks}^{2} \cdot c_{cks} + l_{ckx}^{2} \cdot c_{ckx} + l_{cms}^{2} \cdot c_{cms} + l_{cmx}^{2} \cdot c_{cmx} + r^{2} \cdot c_{zz10} } \right)/4$$。

The matrix ***K*** is:

$$ {\varvec{K}} = \left[ {\begin{array}{*{20}c} {{\varvec{K}}_{{\varvec{1}}} } & {{\varvec{K}}_{{\varvec{3}}} } \\ {{\varvec{K}}_{{\varvec{4}}} } & {{\varvec{K}}_{{\varvec{2}}} } \\ \end{array} } \right] $$

$${\varvec{K}}_{{\varvec{1}}} = \left[ {\begin{array}{*{20}c} 0 & 0 & 0 & 0 & 0 & 0 & 0 \\ 0 & {e^{2} \cdot k_{zz2} } & { - e \cdot k_{zz2} } & 0 & 0 & 0 & 0 \\ 0 & { - e \cdot k_{zz2} } & {K_{3,3} } & {K_{3,4} } & { - k_{zz5} } & {K_{3,6} } & 0 \\ 0 & 0 & {K_{4,3} } & {K_{4,4} } & 0 & {K_{4,6} } & { - k_{zz8} } \\ 0 & 0 & { - k_{zz5} } & 0 & {k_{qpz} + k_{zz5} } & 0 & 0 \\ 0 & 0 & {K_{6,3} } & {K_{6,4} } & 0 & {K_{6,6} } & 0 \\ 0 & 0 & 0 & { - k_{zz8} } & 0 & 0 & {k_{hpz} + k_{zz8} } \\ \end{array} } \right];$$ $${\varvec{K}}_{{\varvec{2}}} = \left[ {\begin{array}{*{20}c} {K_{8,8} } & 0 & 0 & 0 & 0 & 0 \\ 0 & {K_{9,9} } & 0 & 0 & 0 & 0 \\ 0 & 0 & {K_{10,10} } & {K_{10,11} } & 0 & 0 \\ 0 & 0 & {K_{11,10} } & {K_{11,11} } & { - \left( {e \cdot r \cdot k_{zz10} } \right)/2} & 0 \\ 0 & 0 & 0 & { - \left( {e \cdot r \cdot k_{zz10} } \right)/2} & {e^{2} \cdot k_{zz10} } & 0 \\ 0 & 0 & 0 & 0 & 0 & 0 \\ \end{array} } \right];$$

$${\varvec{K}}_{{\varvec{3}}} = \left[ {\begin{array}{*{20}c} 0 & 0 & 0 & 0 & 0 & 0 \\ 0 & 0 & { - \left( {e \cdot r \cdot k_{zz2} } \right)/2} & 0 & 0 & 0 \\ { - k_{zz12} } & 0 & {K_{3,10} } & {K_{3,11} } & 0 & 0 \\ 0 & { - k_{zz13} } & {K_{4,10} } & {K_{4,11} } & { - e \cdot k_{zz10} } & 0 \\ 0 & 0 & 0 & 0 & 0 & 0 \\ 0 & 0 & {K_{6,10} } & {K_{6,11} } & 0 & 0 \\ 0 & 0 & 0 & 0 & 0 & 0 \\ \end{array} } \right];$$ $${\varvec{K}}_{{\varvec{4}}} = \left[ {\begin{array}{*{20}c} 0 & 0 & { - k_{zz12} } & 0 & 0 & 0 & 0 \\ 0 & 0 & 0 & { - k_{zz13} } & 0 & 0 & 0 \\ 0 & { - \left( {e \cdot r \cdot k_{zz2} } \right)/2} & {K_{10,3} } & {K_{10,4} } & 0 & {K_{10,6} } & 0 \\ 0 & 0 & {K_{11,3} } & {K_{11,4} } & 0 & {K_{11,6} } & 0 \\ 0 & 0 & 0 & { - e \cdot k_{zz10} } & 0 & 0 & 0 \\ 0 & 0 & 0 & 0 & 0 & 0 & 0 \\ \end{array} } \right];$$

$$K_{3,3} = k_{cks} + k_{ckx} + k_{cms} + k_{cmx} + k_{zz2} + k_{zz5} + k_{zz12};$$ $$K_{3,4} = k_{cks} + k_{ckx} + k_{cms} + k_{cmx};$$ $$K_{3,6} = - \left( {k_{cks} + k_{ckx} + k_{cms} + k_{cmx} } \right);$$

$$K_{3,10} = \left( {l_{cks} \cdot k_{cks} + l_{ckx} \cdot k_{ckx} + l_{cms} \cdot k_{cms} + l_{cmx} \cdot k_{cmx} + r \cdot k_{zz2} } \right)/2;$$ $$K_{3,11} = \left( {l_{cks} \cdot k_{cks} + l_{ckx} \cdot k_{ckx} + l_{cms} \cdot k_{cms} + l_{cmx} \cdot k_{cmx} } \right)/2;$$

$$K_{4,3} = k_{cks} + k_{ckx} + k_{cms} + k_{cmx};$$ $$K_{4,4} = k_{cks} + k_{ckx} + k_{cms} + k_{cmx} + k_{zz8} + k_{zz10} + k_{zz13};$$ $$K_{4,6} = - \left( {k_{cks} + k_{ckx} + k_{cms} + k_{cmx} } \right);$$

$$K_{4,10} = \left( {l_{cks} \cdot k_{cks} + l_{ckx} \cdot k_{ckx} + l_{cms} \cdot k_{cms} + l_{cmx} \cdot k_{cmx} } \right)/2;$$ $$K_{4,11} = \left( {l_{cks} \cdot k_{cks} + l_{ckx} \cdot k_{ckx} + l_{cms} \cdot k_{cms} + l_{cmx} \cdot k_{cmx} + r \cdot k_{zz10} } \right)/2;$$

$$K_{6,3} = - \left( {k_{cks} + k_{ckx} + k_{cms} + k_{cmx} } \right);$$ $$K_{6,4} = - \left( {k_{cks} + k_{ckx} + k_{cms} + k_{cmx} } \right);$$ $$K_{6,6} = k_{cks} + k_{ckx} + k_{cms} + k_{cmx};$$

$$K_{6,10} = - \left( {l_{cks} \cdot k_{cks} + l_{ckx} \cdot k_{ckx} + l_{cms} \cdot k_{cms} + l_{cmx} \cdot k_{cmx} } \right)/2;$$ $$K_{6,11} = - \left( {l_{cks} \cdot k_{cks} + l_{ckx} \cdot k_{ckx} + l_{cms} \cdot k_{cms} + l_{cmx} \cdot k_{cmx} } \right)/2;$$

$$K_{8,8} = k_{qdm} + k_{qdz} + k_{zz12};$$ $$K_{9,9} = k_{hdm} + k_{hdz} + k_{zz13};$$ $$K_{10,3} = \left( {l_{cks} \cdot k_{cks} + l_{ckx} \cdot k_{ckx} + l_{cms} \cdot k_{cms} + l_{cmx} \cdot k_{cmx} + r \cdot k_{zz2} } \right)/2;$$

$$K_{10,4} = \left( {l_{cks} \cdot k_{cks} + l_{ckx} \cdot k_{ckx} + l_{cms} \cdot k_{cms} + l_{cmx} \cdot k_{cmx} } \right)/2;$$ $$K_{10,6} = - \left( {l_{cks} \cdot k_{cks} + l_{ckx} \cdot k_{ckx} + l_{cms} \cdot k_{cms} + l_{cmx} \cdot k_{cmx} } \right)/2$$

$$K_{10,10} = \left( {l_{cks}^{2} \cdot k_{cks} + l_{ckx}^{2} \cdot k_{ckx} + l_{cms}^{2} \cdot k_{cms} + l_{cmx}^{2} \cdot k_{cmx} + r^{2} \cdot k_{zz2} } \right)/4;$$ $$K_{10,11} = \left( {l_{cks}^{2} \cdot k_{cks} + l_{ckx}^{2} \cdot k_{ckx} + l_{cms}^{2} \cdot k_{cms} + l_{cmx}^{2} \cdot k_{cmx} } \right)/4;$$

$$K_{11,3} = \left( {l_{cks} \cdot k_{cks} + l_{ckx} \cdot k_{ckx} + l_{cms} \cdot k_{cms} + l_{cmx} \cdot k_{cmx} } \right)/2;$$ $$K_{11,4} = \left( {l_{cks} \cdot k_{cks} + l_{ckx} \cdot k_{ckx} + l_{cms} \cdot k_{cms} + l_{cmx} \cdot k_{cmx} + r \cdot k_{zz10} } \right)/2;$$

$$K_{11,6} = - \left( {l_{cks} \cdot k_{cks} + l_{ckx} \cdot k_{ckx} + l_{cms} \cdot k_{cms} + l_{cmx} \cdot k_{cmx} } \right)/2;$$ $$K_{11,10} = \left( {l_{cks}^{2} \cdot k_{cks} + l_{ckx}^{2} \cdot k_{ckx} + l_{cms}^{2} \cdot k_{cms} + l_{cmx}^{2} \cdot k_{cmx} } \right)/4;$$

$$K_{11,11} = \left( {l_{cks}^{2} \cdot k_{cks} + l_{ckx}^{2} \cdot k_{ckx} + l_{cms}^{2} \cdot k_{cms} + l_{cmx}^{2} \cdot k_{cmx} + r^{2} \cdot k_{zz10} } \right)/4$$。

The matrix ***Z*** is:

$$ {\varvec{Z}} = \left[ {\beta_{z1} ,\gamma_{z2} ,z_{3} ,z_{7} ,z_{5} ,z_{6} ,z_{8} ,z_{12} ,z_{13} ,\eta_{z3} ,\eta_{z7} ,\gamma_{z10} ,\beta_{z11} } \right]^{T} $$

The matrix ***F*** is:

$$ {\varvec{F}} = \left[ {R_{z1} p\cos \alpha_{z1} + R_{x1} p\sin \alpha_{z1} ,d_{xy} k_{zz2} e/2, - d_{xy} k_{zz2} /2, - d_{xy} k_{zz10} /2,0,0,0,} \right.\left. {w_{xd} k_{qdz} /2,w_{xd} k_{hdz} /2, - d_{xy} k_{zz2} r/4, - d_{xy} k_{zz10} r/4,d_{xy} \cdot k_{zz10} \cdot e/2,0} \right]^{T} .$$
